# Cs and Br tuning to achieve ultralow-hysteresis and high-performance indoor triple cation perovskite solar cell with low-cost carbon-based electrode

**DOI:** 10.1016/j.isci.2024.109306

**Published:** 2024-02-22

**Authors:** Ladda Srathongsian, Anusit Kaewprajak, Atittaya Naikaew, Chaowaphat Seriwattanachai, Napan Phuphathanaphong, Anuchytt Inna, Thana Chotchuangchutchaval, Woraprom Passatorntaschakorn, Pisist Kumnorkaew, Somboon Sahasithiwat, Duangmanee Wongratanaphisan, Pipat Ruankham, Ratchadaporn Supruangnet, Hideki Nakajima, Pasit Pakawatpanurut, Pongsakorn Kanjanaboos

**Affiliations:** 1 School of Materials Science and Innovation, Faculty of Science, Mahidol University, Nakhon Pathom 73170, Thailand; 2 National Nanotechnology Center (NANOTEC), National Science and Technology Development Agency, Pathum Thani 12120, Thailand; 3 Center of Sustainable Energy and Engineering Materials (SEEM), College of Industrial Technology, King Mongkut’s University of Technology North Bangkok, Bangkok 10800, Thailand; 4 Department of Mechanical Engineering Technology, College of Industrial Technology, King Mongkut’s University of Technology North Bangkok, Bangkok 10800, Thailand; 5 Department of Physics and Materials Science, Faculty of Science, Chiang Mai University, Chiang Mai 50200, Thailand; 6 National Metal and Materials Technology Center (MTEC), National Science and Technology Development Agency, Pathum Thani 12120, Thailand; 7 Synchrotron Light Research Institute (Public Organization), Nakhon Ratchasima 30000, Thailand; 8 Department of Chemistry and Center of Sustainable Energy and Green Materials, Faculty of Science, Mahidol University, Bangkok 10400, Thailand; 9 Center of Excellence for Innovation in Chemistry (PERCH CIC), Ministry of Higher Education, Science, Research and Innovation, Bangkok 10400, Thailand

**Keywords:** Devices, Energy materials, Materials science

## Abstract

With high efficacy for electron-photon conversion under low light, perovskite materials show great potential for indoor solar cell applications to power small electronics for internet of things (IoTs). To match the spectrum of an indoor LED light source, triple cation perovskite composition was varied to adjust band gap values via Cs and Br tuning. However, increased band gaps lead to morphology, phase instability, and defect issues. 10% Cs and 30% Br strike the right balance, leading to low-cost carbon-based devices with the highest power conversion efficiency (PCE) of 31.94% and good stability under low light cycles. With further improvement in device stack and size, functional solar cells with the ultralow hysteresis index (HI) of 0.1 and the highest PCE of 30.09% with an active area of 1 cm^2^ can be achieved. A module from connecting two such cells in series can simultaneously power humidity and temperature sensors under 1000 lux.

## Highlights

• Triple cation perovskite with 10% Cs and 30% Br is fit for collecting indoor light
• High Cs/Br leads to wrinkle morphology and poor interface between perovskite and HTL
• Carbon-based solar cell with ultralow HI of 0.1 and PCE of 30.09% can be achieved
• Connecting 2 devices (1 cm^2^ active area) in series can power IoTs under 1000 lux

## Graphical abstract

**Figure:**
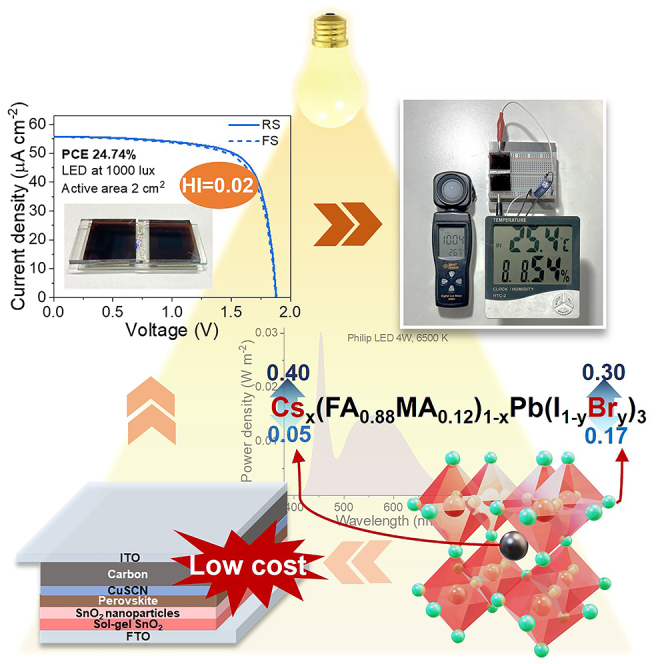

## Introduction

Perovskite solar cells (PSCs) have been improving significantly for a decade; now the device reached 26.1% efficiency for outdoor applications (AM1.5G).^1^ In addition, PSCs can be used under low intensity because of its good absorption coefficient, band gap tunability, and low trap density,^2^^,^^3^^,^^4^ expanding the applicability of solar cells for internet of things (IoTs). Nowadays the IoTs market has grown exponentially, leading to an increased number of small electronic devices in smart home/buildings. Indoor solar cells can be an alternative energy source for such devices. Although the power density from indoor light sources is ∼300 times less than that of sunlight, it is sufficient to power IoTs sensors.^5^

There are many approaches to enhance indoor performance by improving interfaces between different layers within PSCs. For perovskite/electron transport layer (ETL), Li et al. passivated the interface between wide-band gap perovskite and ETL by using phenethylammonium halides, resulting in reduction of voltage loss and phase segregation.^6^ Furthermore, Xu et al. demonstrated the use of alkali-fluoride as inorganic walls on both sides of perovskite layer to passivate surface defects and physically prevent oxygen and moisture, resulting in high efficiency and stable solar cell device.^7^

Moreover, the band gap energy (E_g_) of perovskite plays an important role for indoor performance. From the Shockley-Queisser limit calculation based on the indoor light spectrum, a maximum PCE of 57% can be obtained by optimal band gaps between 1.8 and 1.9 eV; the calculation was demonstrated for different indoor light sources such as 3000–6500 K LEDs, 2700–6500 K fluorescent lamps, and white organic LEDs at 3360 K and 4240 K.^8^^,^^9^ For perovskite materials, composition tuning is the easiest way to achieve the required band gap; there are numerous reports exhibiting the effects of perovskite composition on its properties and performances.^10^^,^^11^^,^^12^^,^^13^^,^^14^^,^^15^^,^^16^ To achieve large band gaps, halide is the most popular tuning site, as it strongly affects electronic band.^17^ Lim et al. demonstrated the effect of bromide doping in MAPbI_3_ for indoor performance. With the mixed halide MAPb(I_0.9_Br_0.1_)_3_, an indoor PCE of 36.2% was achieved.^18^ Similarly, Singh et al. studied the effects of MAPb(I_1-x_Br_x_)_3_ crystal structure on indoor performance. MAPbBr_3_ provided the high V_oc_ of 1.15 V under indoor light.^19^ Cheng et al. used triple anions (MAPbI_2-x_BrCl_x_) to match the fluorescent spectrum, achieving an indoor PCE of 36%.^20^ Moreover, there are other studies of bromide or chloride doping on mixed cation perovskite for indoor applications such as FAMA,^21^ MACs,^22^^,^^23^ and CsFAMA.^24^^,^^25^ Mostly, the proper bromine ratio in halide site is 10%–30%. Although we can achieve a wide-band gap with a high Br amount, the mixed halide perovskite suffers from high trap density^18^ and phase segregation of iodide-rich and bromide-rich phases, as reported by Hoke et al.^26^ This effect suppresses device performance by reducing V_oc_ due to potential losses when charge carriers move from large to small band gap sites. In addition, the photogenerated charge carriers can be eliminated due to the recombination within the iodide-rich phase.^27^^,^^28^

Only a few reports pay attention to the cation site of perovskite materials for low light applications. For example, Singh et al. investigated the suitable ratio of MA/FA and found that with a quasi-cubic structure of 10% MA/FA exhibits the highest indoor PCE of 34.07%.^29^ For outdoor solar cell application, triple cation perovskite CsFAMA has been developed due to its high efficiency and stability.^30^ In many cases, Cs cation was added as the stabilizer for perovskite structure due to Gibbs free energy and crystallization temperature reduction.^31^ Moreover, Svanström et al. found that Cs reduces light induced phase segregation in wide-band gap CsFA and CsMA perovskites, when compared to FAMA.^32^ Besides, the ratios of Cs cation and halide ions are also important. Rehman et al. found Cs20 (20% Cs, 80% FA) shows high photostability for CsFAPb(I_0.83_Br_0.17_)_3_ under 1-sun.^33^ Bush et al. reported that slightly more Cs than Br suppresses halide segregation and improves the photostability in CsFA perovskite^34^ for sunlight usage.

Morphology is another critical parameter for PSC devices. Smooth surface and large grain size lead to high quality perovskite films and therefore enhance performance and stability.^35^ There are lots of factors that affect perovskite morphology such as solvent type, anti-solvent type, film fabrication procedure, and compositions, as these factors control the drying process. Bush et al. and Kim et al. demonstrated that wrinkle patterns on perovskite films can be caused by different deposition methods and solvents.^36^^,^^37^ Moreover, Bercegol et al. found that the Cs component strongly influences wrinkle density on the perovskite surface. Besides, Cs-rich was observed at the hill of the wrinkle, indicating inhomogeneous Cs distribution.^38^ Although rough surface poorly affects device performances, Braunger et al. showed that the proper composition (optimized Cs and Br) with small wrinkles can improve the performance by increasing of J_sc_ and V_oc_.^39^

In order to study the effects of perovskite composition in terms of both cation and anion sites for indoor light application, we investigated the triple cation perovskite system Cs_x_(FA_0.88_MA_0.12_)_1-x_Pb(I_1-y_Br_y_)_3_ with the band gaps in the range of 1.6–1.7 eV. Cs is varied from 5%, 10%, 20%, 30%, and 40%, namely Cs5, Cs10, Cs20, Cs30, and Cs40, respectively, while Br is either 17% or 30% (Br17 or Br30) to provide insights regarding compositional effects on morphology, defect, stability, and resulting solar performances.

## Results and discussion

**Figure 1:**
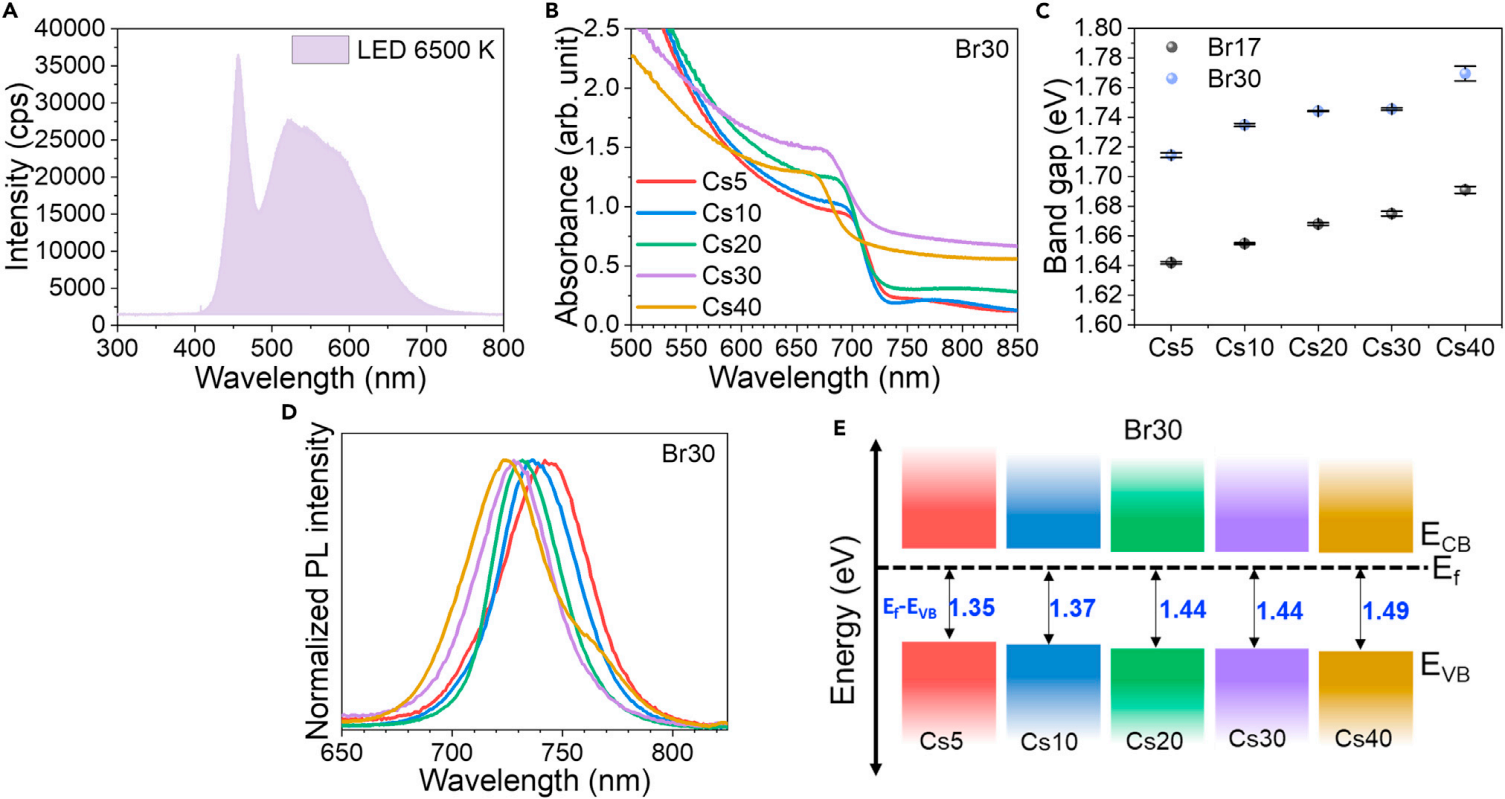
Optical and electronic properties (A) Spectrum of LED light (Phillip E27, LED 4W, 6500K). (B) Absorbance spectra of Br30 with 5–40% Cs. (C) Band gap energies of Br17 and Br30 systems with 5–40% Cs. The error bar indicates uncertainty in band gap fitting. (D) Normalized PL spectra of Br30 with 5–40% Cs. (E) Energy band diagram of Br30 perovskite films with 5–40% Cs with respect to E_f_ (E_f_ = 0).

Figure 1A presents the spectrum of LED 6500 K, which is one of the most common LED indoor light sources nowadays and is used for performance and stability study in this work. The spectrum covers wavelength from 400 nm to 700 nm, while standard AM1.5G covers much wider spectrum range. Consequently, the optimal band gap of absorber materials for indoor light is different and located at 1.8–1.9 eV.^8^^,^^9^ The absorbance spectra of the perovskite films are shown in Figure 1B. For Br30, the spectra shift to shorter wavelengths and therefore wider band gap energy with an increasing amount of Cs. Moreover, a similar trend is observed with Br17 in Figure S1A. As identified via the Tauc plot for Cs variation, the band gaps were enlarged from 1.64 to 1.69 eV for Br17 and 1.71 to 1.77 eV for Br30 (Figure 1C). This tunability potentially benefits indoor light performance by matching the indoor light spectrum.^8^^,^^20^ Interestingly, the spectra of Cs20 exhibit the highest absorbance values in both the Br17 and Br30 systems. On the other hand, high absorbance intensities at the infrared region of Cs30 and Cs40 can be linked to more light scattering from inhomogeneous surface and wrinkle-like topography.^40^ For the photoluminescence (PL) spectra in Figure 1D (Br30 system) and Figure S1B (Br17 system), the blue shifting trend was similarly observed with an increased Cs amount. However, Cs40Br30 exhibits phase segregation, having an extra iodide-rich phase as the second peak at 770 nm, which can lower device performance due to charge carrier trapping and more recombination within the iodide-rich phase.^41^^,^^42^^,^^43^^,^^44^ Additionally, X-ray fluorescence (XRF) results confirmed that we successfully incorporated Cs and Br in the perovskite structure (Figure S1C). Furthermore, we performed ultraviolet photoelectron spectroscopy (UPS) measurement to understand the effect of the composition on the electronic band structure of perovskite materials. Figure 1E represents the calculated valence band maxima (VBM) and conduction band minima (CBM), with respect to zero fermi energy (E_f_) of the Br30 system with Cs concentration from 5 to 40%. As Cs concentration increases, VBM shifts downward to more negative energy. A previous report explained that the shifting of VBM enhances the driving force for holes to hole transport layer (HTL), leading to higher V_oc_.^45^

**Figure 2:**
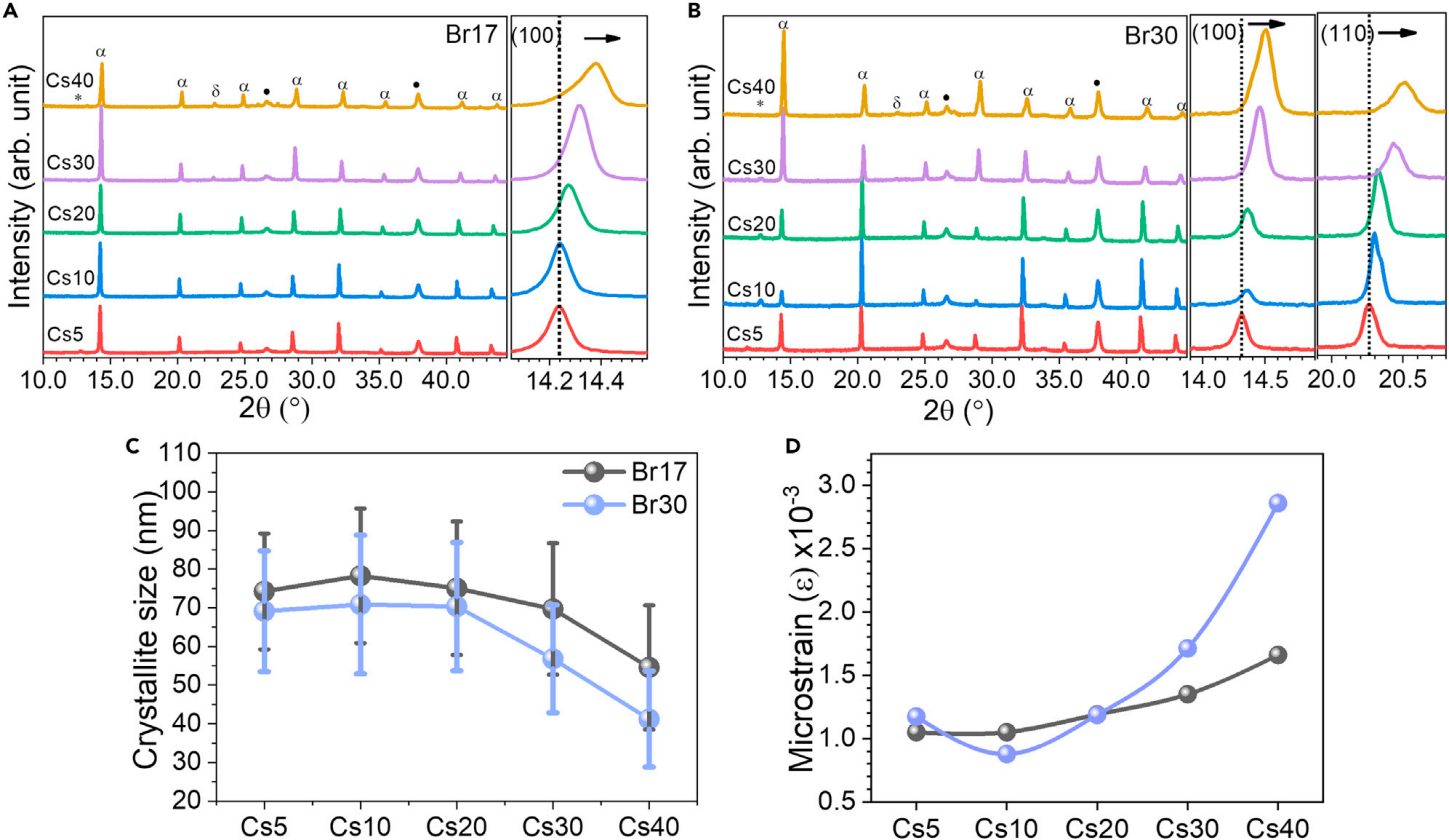
Crystal structure and microstrain analysis XRD spectra of (A) Br17 and (B) Br30 perovskite films with 5–40% Cs (α = perovskite peak, δ = yellow phase of CsPbI_3_, ∗ = PbI_2_, ⋅ = FTO substrate). (C) Crystallite size of perovskite films from Br17 and Br30 systems (average values from (100), (110), (200), and (220) planes). The error bar indicates standard deviations of crystallite size values from different XRD planes. (D) Microstrain of perovskite films from Br17 and Br30 systems (calculated by using the Williamson-Hall equation).

Furthermore, the effect of composition on the crystallinity of perovskite films was investigated by using the XRD technique. Figures 2A and 2B show XRD characteristic peaks of perovskite materials for all conditions. The main peaks at 14.2°–14.5° and 20.2°–20.4° correspond to the (100) and (110) crystal planes, respectively.^32^ With increasing Cs amount, the perovskite peaks shift toward higher 2θ, indicating a decreasing lattice parameter due to the smaller radius of Cs. Moreover, the Br30 samples (Cs5 - Cs20) exhibit preferential orientation for the (110) plane instead of (100).^46^ Cs40 samples for both systems have an inactive phase of CsPbI_3_ at 22.8°,^47^ indicating poor photoactive layers. Additionally, the crystallite size of each composition was calculated using Scherrer’s equation; the larger crystallite size can be obtained with small amounts of Br and Cs, while the higher amounts lead to small size as seen in Figure 2C which is in agreement with other reports.^48^^,^^49^ Microstrain of perovskite lattice was further explored by the Williamson-Hall plot (Figure S2) as follows:

(Equation 1) $$\beta \;\cos\;\theta =4\varepsilon \;\sin\;\theta +\frac{K\lambda }{D}$$

where β is full width at half maximum of the diffraction peak (radian), θ is diffraction angle (degree), λ is X-ray wavelength (nm), K is shape factor (0.9), and D is crystallite size of the perovskite crystal (nm). Cs10Br30 shows lowest microstrain, while higher Br or Cs content exhibits higher strain, especially in the Br30 perovskite system as shown in Figure 2D due to homogenization effect of Br.^50^ Previous studies also reported that lattice strain increases defect density and non-radiative recombination, resulting in poor device performance and stability.^51^^,^^52^^,^^53^

**Figure 3:**
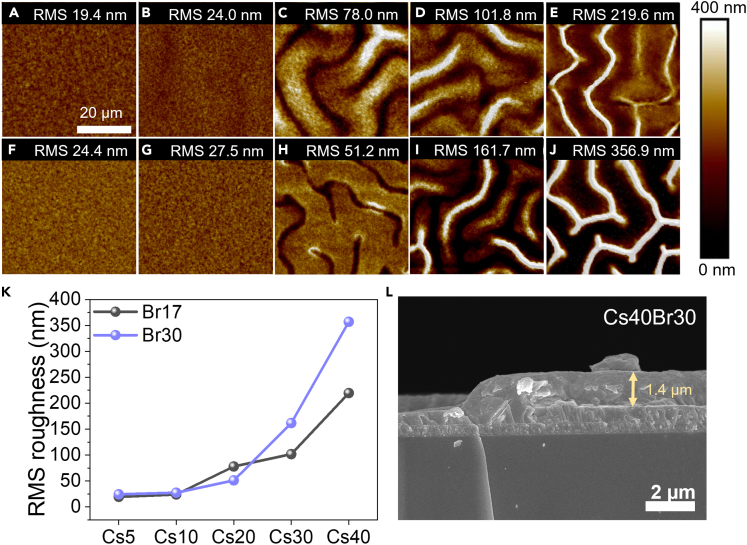
Perovskite morphology (A–E) AFM topography images of Br17 perovskite films with Cs5, Cs10, Cs20, Cs30, and Cs40, respectively. (F–J) AFM topography images of Br30 perovskite films with Cs5, Cs10, Cs20, Cs30, and Cs40, respectively. (K) Root mean square (RMS) roughness of Br17 and Br30 perovskite films with Cs5, Cs10, Cs20, Cs30, and Cs40, respectively. (L) SEM cross-sectional image of Cs40Br30.

Figures 3A–3J present the surface topography of each composition from atomic force microscopy (AFM), the surface roughness increases with higher amount of Br and Cs as shown in Figure 3K. Obviously, the wrinkle patterns appear with more than 10% Cs inclusion, and the wrinkle amplitude is highest with Cs40 as shown in Figure S3. Cs strongly influences wrinkle density. The wrinkle patterns are caused by the stress/strain relaxation of perovskite films during the crystallization process.^36^ Normally, the relaxation is governed by various factors such as solvent, anti-solvent, deposition procedure, and film/substrate lattice mismatch.^36^^,^^37^ In this case, the low crystallization temperature of Cs accelerates the transition between the intermediate phase and the perovskite crystal.^36^^,^^54^ Moreover, the solubility of Cs in the form of CsPbI_3_ in DMF, which accounts for 80% of the perovskite precursor solvent, is low compared to FAPbI_3_, FAPbBr_3_, and MAPbBr_3_,^55^^,^^56^^,^^57^ hence fast crystallization can be observed with increased Cs, leading to a rough surface with a wrinkle pattern. This result is consistent with a previous report.^39^ Besides, the Br amount also affects the film roughness by exhibiting faster crystallization when compared to that of I as reported in another work.^58^ Morphology and grain size were examined by scanning electron microscopy (SEM) (Figures S4 and S5); Br30 samples show slightly larger grain size of 244–280 nm with a large distribution, while Br17 samples have an average grain size of 211–220 nm with a narrow distribution. Moreover, SEM images of surface wrinkles (similarly seen in the AFM results) for Cs20, Cs30, and Cs40 systems are as shown in Figure S6. Apparently, Cs40 shows over 1 μm thickness at the wrinkle hills (Figure 3L), which may affect the interface between perovskite and charge transport layer.

With further characterization by AFM in the conductive mode (C-AFM), we studied localized current maps of perovskite surfaces under low light excitation while applying no bias to get generated currents at different locations (I_sc_ map) and 0.5 V in the forward condition to gain insights about V_oc_ landscape (V_oc_ map). Figures S7A–S7E illustrates localized I_sc_ maps of the Br30 system. The currents increase with high Cs concentration as a result of the matching between the light source and material absorbance spectra. Moreover, a similar trend was observed on current mapping at 0.5 V (the V_oc_ condition) as shown in Figures S7F–S7J where increasing current is related to higher V_oc_ due to the larger band gap with the increasing of Cs concentration.^59^ Although Cs40 has the most absorbance spectrum matching with the indoor light source, the smallest crystallite size along with the inactive phase of CsPbI_3_ leads to a decrease of charge carriers and therefore low photocurrent. Apparently, C-AFM images demonstrate grain features along with the current distribution. Most of the bright spots, which represent high current, appear at the larger grains due to their high crystallinity and low trap states^35^ while the dark spots are shown at the smaller grains or non-photoactive residues such as PbI_2_.^60^^,^^61^ Moreover, the high current at the grain boundaries (GBs) at 0 V bias suggests that GBs has low defects and dislocations, becoming highways for charge movement.^60^^,^^62^^,^^63^

**Figure 4:**
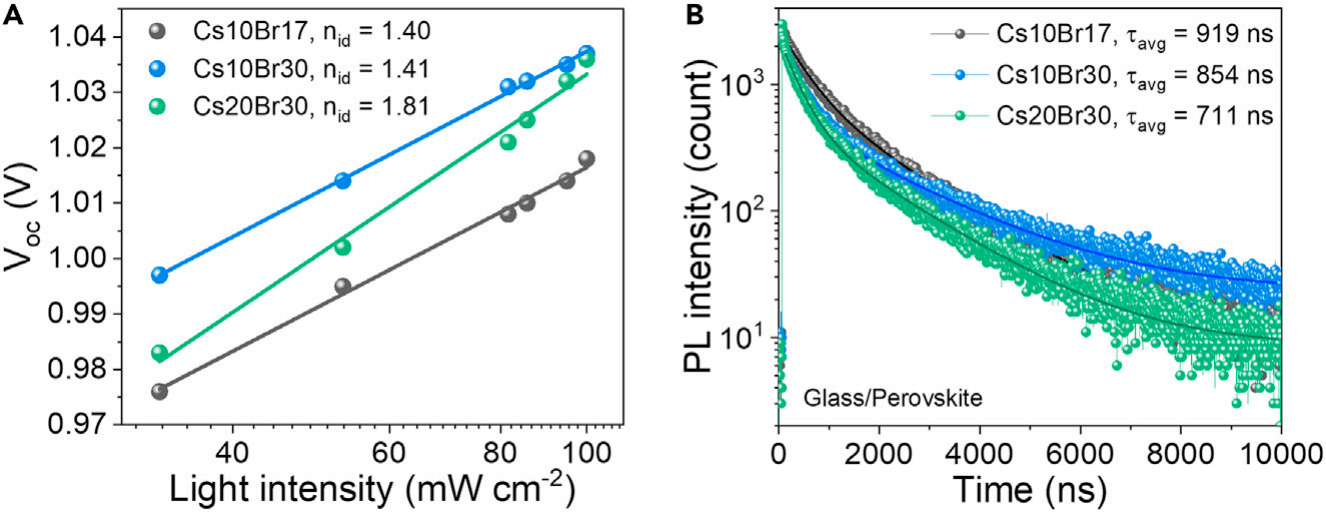
Defect investigation of perovskite films and devices (A) V_oc_-Light intensity dependence of Cs10Br17, Cs10Br30, and Cs20Br30 devices. (B) PL lifetimes of Cs10Br17, Cs10Br30, and Cs20Br30 perovskite films on glass.

To understand recombination mechanisms of different compositions, we performed V_oc_-Light intensity dependence as shown Figure 4A. From the equation V_oc_ ≈ n_id_kTln(L)/q, the ideality factor (n_id_) is related to recombination process; n_id_ = 1 refers to band-to-band recombination, while n_id_ = 2 refers to trap assisted recombination or Shockley-Read-Hall (SRH) recombination.^64^^,^^65^^,^^66^ In Figure 4A, Br30 samples with both 10% and 20% Cs obviously show higher V_oc_ values at the light intensity when compared to those of Br17 due to wider band gaps.^66^ In terms of calculated n_id_, Cs10Br17 and Cs10Br30 have lowest n_id_ of 1.40 and 1.41, respectively, suggesting less SRH recombination, while Cs20Br30 has n_id_ values of 1.81; the n_id_ value close to 2 for Cs20Br30 indicates high trap-assisted recombination. As the space-charge regions also have impact on ideality factor (n_id_), we further performed impedance spectroscopy of the solar cell devices. The capacitance at low frequency represents charge accumulation at the interface, leading to space-charge regions.^67^ Cs20Br30 exhibits high capacitance when compared to those of Cs10Br17 and Cs10Br30 as seen in Figure S8. Theoretically, the high capacitance should lower ideality factor (n_id_) due to space-charge region at the interface;^65^ however, Cs20Br30 achieves the highest n_id_ value (Figure 4A), suggesting that the effect of space-charge region is less than that of perovskite compositions. The charge carrier lifetime of perovskite films on glass substrate is presented in Figure 4B and Table S1. The parameters were obtained by using a biexponential fitting; I(t) = A_1_exp(-t/τ_1_) + A_2_exp(-t/τ_2_), where τ_1_ is the fast decay component which is associated with non-radiative recombination at the interface and surface and τ_2_ is the slow decay component which relates to intrinsic band-to-band radiative recombination in bulk,^68^ A_1_ and A_2_ are weights of the decay components τ_1_ and τ_2_. Cs10Br17 shows the longest τ_avg_ of 919 ns with the lowest fast decay weight component (A_2_), indicating high intrinsic recombination and less defects at the surface. Although Cs10Br30 exhibits a slightly lower τ_avg_ of 854 ns, it shows the longest slow decay (τ_2_), suggesting high-quality crystal formation with low defect density. Cs20Br30 has the shortest τ_avg_ of 711 ns with the highest weight of the fast decay component (A_1_), pointing to high non-radiative recombination, consistent with the high ideality factor value in Figure 4A. Furthermore, we fabricated electron-only device with the configuration FTO/SnO_2_/Perovskite/PCBM/Carbon for space-charge limited current (SCLC) measurement. Trap density is determined from trap filled limited voltage (V_TFL_) in Figure S9 via the following equation: N_trap_ = 2ϵϵ_0_V_TFL_/qd^2^^.^ Although V_TFL_ values increase with higher Cs concentration, valleys and hills from the wrinkle feature for the case of high Cs concentration cause unreliable thickness estimation and trap density values.^69^ The perovskite thicknesses were measured by SEM as shown in Table S2.

**Figure 5:**
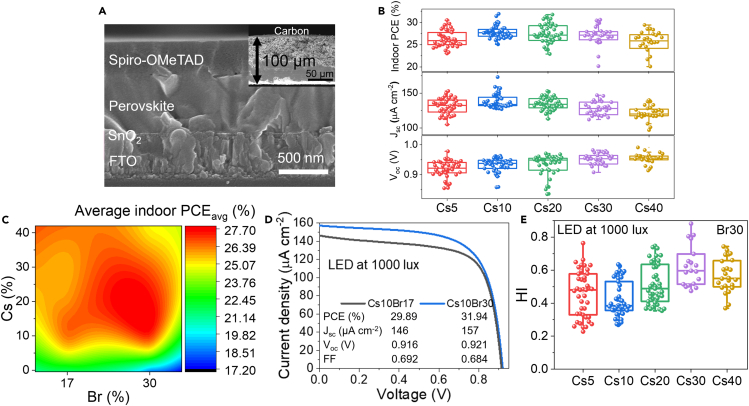
Perovskite device and performance (A) Cross-sectional SEM image of solar cell device with configuration FTO/SnO_2_/Perovskite/Spiro-OMeTAD/Carbon (inset shows a cross-section image of the carbon sheet). (B) Device performance parameters (PCE, J_sc_, and V_oc_) under indoor light at 1000 lux. (C) Contour map of average indoor solar cell performances for different Br and Cs concentrations. (D) J-V graph of Cs10Br17 and Cs10Br30 (reverse scan). (E) Hysteresis index of Br30 with 5–40% Cs.

To investigate device performance, we fabricated solar cell devices with the structure FTO/SnO_2_/Perovskite/Spiro-OMeTAD/Carbon as shown in SEM cross-sectional image in Figure 5A. Indoor light performance was performed under LED at 1000 lux (P_in_ = 0.31 mW cm^−2^). Figure 5B shows boxplots of indoor performance for Br30 system. Cs10 shows the maximum J_sc_ while higher Cs concentration reduces J_sc_. This result contrasts with localized I_sc_ mapping from conductive AFM as the J_sc_ value is from the whole device which contains information from many layers and much larger area, therefore micron-sized surface wrinkle should be considered. The very rough surface of high Cs perovskite affects homogeneity and coverage of hole transport layer as shown in Figure S10, resulting in the reduction of J_sc_.^70^ Similar to the V_oc_ map from AFM, V_oc_ increases with higher Cs concentration due to larger band gap induced by small Cs. As higher V_oc_ can be achieved by two independent means (enlarging band gaps and lowering trap states), the positive trend indicates stronger band gap dependence when compared to surface defects from wrinkles. The Cs10 condition does not have any wrinkle feature, leading to the highest average indoor PCE of 27.7%. In addition, the different composition show similar fill factor (FF), as shown in Figure S11. To clearly illustrate the effect of Cs/Br composition on indoor solar performances, the contour map is presented in Figure 5C; the red regime depicts optimal compositions with peak indoor conversion capacity. This result is consistent with other reports that wide-band gap materials around 1.8–1.9 eV are suitable for indoor light source.^8^ Therefore, the indoor PCE (champion device) of Cs10Br30 is higher than that of Cs10Br17 as presented in Figure 5D. On the other hand, there is no significant difference of PCE from different compositions for both Br17 and Br30 system under 1-sun as shown in Figure S12. Under 1-sun, J_sc_ and external quantum efficiency (EQE) decrease with increased Cs concentration/band gap (Figure S13) due to incompatible absorption edge while V_oc_ becomes higher with larger band gap; thus, the similar PCE was obtained due to the trade-off between J_sc_ and V_oc_. Moreover, the optical loss was investigated from transmittance of substrate FTO/SnO_2_ as shown in Figure S13, the highest transmittance of 85% is similar to the maximum EQE values, suggesting the substrate as the main source of the optical loss. Hysteresis stems from the PCE differences between forward and reverse scans due to charge trapping, ion migration, and capacitive effect.^71^^,^^72^ Perovskite solar cell under indoor light typically suffers from large hysteresis when compared to the outdoor solar cells as shown in Figure S14 in agreement with another report.^73^ Figure 5E illustrates hysteresis index (HI) of Br30 system with different Cs concentrations; HI is lowest with Cs10 and then increases with higher Cs, indicating more ion migration and defects, consistent with the observed trends in microstrain and ideality factor as illustrated in Figures 2, 4, and S15.

**Figure 6:**
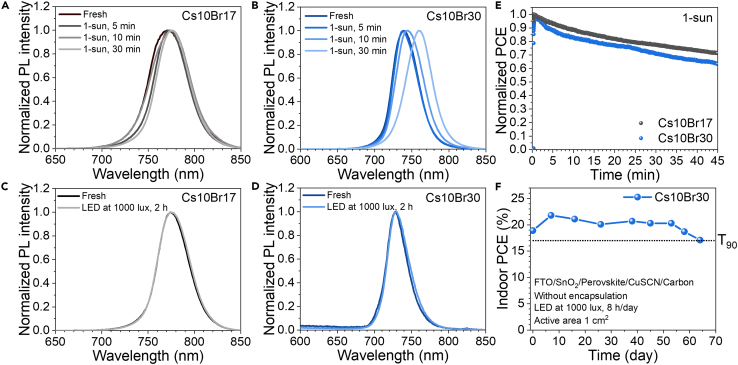
Stability of perovskite thin films and devices (A and B) Normalized PL spectra of Cs10Br17 and Cs10Br30 after soaking in 1-sun for 5–30 min. (C and D) Normalized PL spectra of Cs10Br17 and Cs10Br30 after soaking in LED at 1000 lux for 2 h. (E) MPPT of Cs10Br17 and Cs10Br30 devices under 1-sun illumination. (F) Stability test of the Cs10Br30 device in ambient air (50–80% RH) under 1000 lux illumination for 8 h/day without encapsulation.

For mixed halide perovskite with Br over 20%, light-induced phase segregation according to the Hoke effect should be considered.^26^^,^^74^ Accordingly, the PL comparison between Cs10Br17 and Cs10Br30 was made after 1-sun and LED light soaking, as shown in Figures 6A–6D. Under 1-sun, the Cs10Br30 peak shifts toward a higher wavelength after soaking for 30 min, suggesting phase segregation toward the iodide-rich phase;^74^ however, the Cs10Br17 peak is slightly red-shifted, indicating materials stability with a lower Br content. In case of LED light soaking, there is no phase segregation for both Cs10Br17 and Cs10Br30 after 2 h soaking. In addition, the PL spectrum of Cs20Br30 in Figure S16 also shows the same results under LED soaking.

The LED stability implies that the developed wide-band gap materials can be used for indoor applications, in agreement with the previous report.^25^ To check device stability, Figure 6E illustrates maximum power point tracking (MPPT) comparison between Cs10Br17 and Cs10Br30 under 1-sun for 45 min. Normalized PCE of Cs10Br17 is more stable than Cs10Br30, consistent with the light-induced phase segregation experiment. To investigate the device stability in a real indoor situation, a 1 cm^2^ device of Cs10Br30 perovskite without encapsulation was fabricated and stored under indoor light at 1000 lux for 8 h/day in ambient air. The indoor PCE remains 90% after 60 days of testing, as shown in Figure 6F. Hence, our material design provides promising indicators, as seen in both performance and stability for indoor light applications.

**Figure 7:**
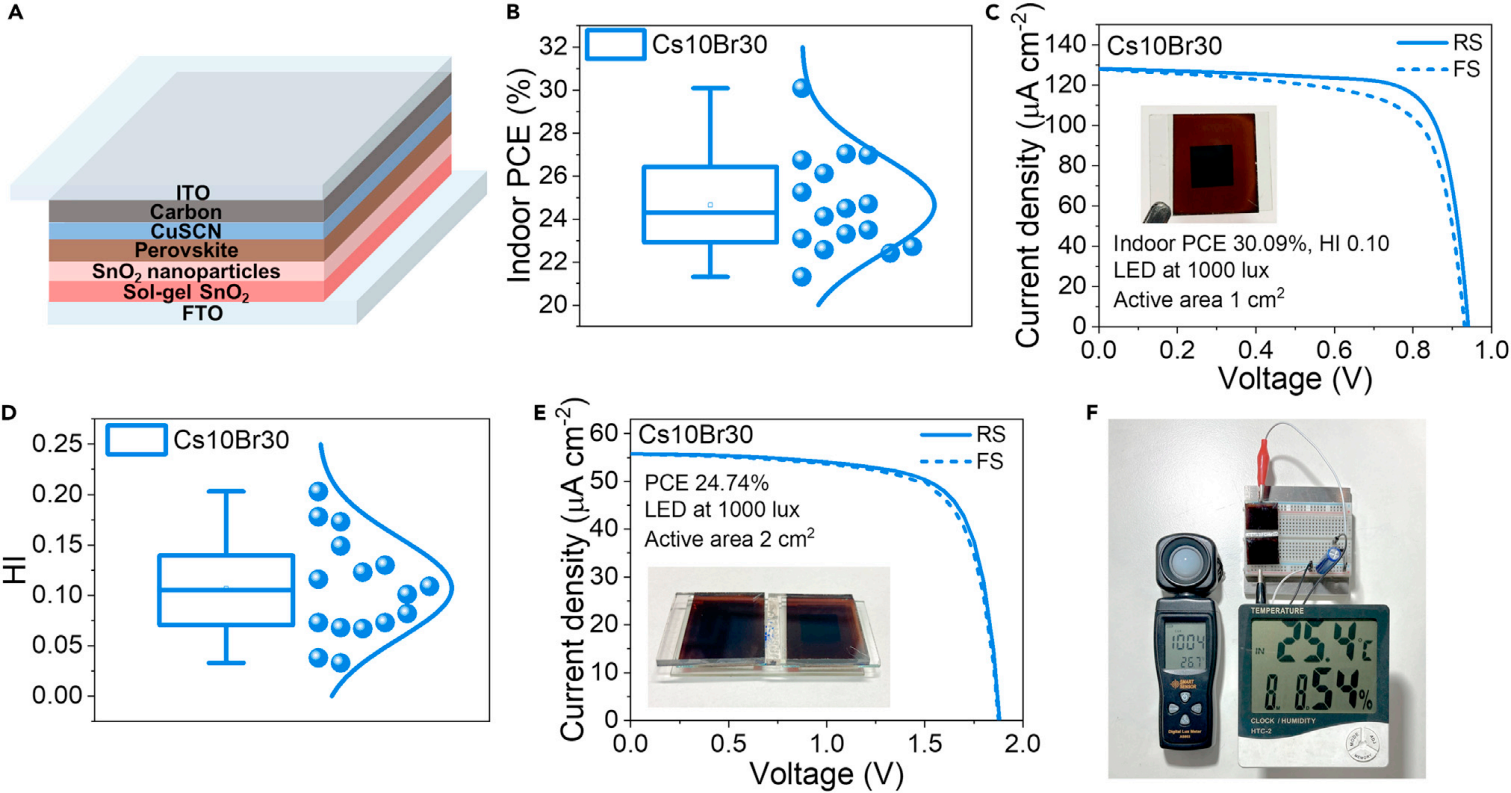
Improved perovskite structure and perovskite module demonstration (A) Schematic of the device structure with configuration FTO/Sol-gel SnO_2_/SnO_2_ NPs/Perovskite/CuSCN/Carbon/ITO. (B) Indoor PCE statistics of Cs10Br30. (C) J-V graph of the champion device (Cs10Br30). (D) HI statistical data of Cs10Br30. (E) J-V graphs of two Cs10Br30 devices in series with a total active area of 2 cm^2^ under LED at 1000 lux. (F) Photograph of thermo-hygrometer (HTC-2 model) powered by the indoor solar cell under 1004 lux.

As HI is also potently influenced by ETL and HTL,^73^^,^^75^ we further optimized the device stack by utilizing bilayer ETL (SnO_2_ nanoparticles (SnO_2_ NPs) on top of sol-gel SnO_2_), leading to the improvement of ETL morphology and the interface between ETL and perovskite;^76^^,^^77^ HTL was also changed to be couper(I) thiocyanate (CuSCN) due to its better energy level alignment with perovskite material, resulting in a more efficient hole extraction and reduced charge recombination and accumulation at the interface.^78^^,^^79^ With improved ETL and HTL, the ultralow-hysteresis and high-performance carbon-based perovskite solar cell for indoor usage can be achieved (Figure 7A). The solar devices with 1 cm^2^ active area achieve the highest indoor PCE of 30.09% and average PCE of 24.66% along with ultralow average HI of around 0.1 in Figures 7B–7D and Table S3, significantly below HI of 0.4 from the previous device configuration in Figure 5E. Our HI values are comparable to those reported for indoor perovskite solar cells with metal electrodes (Table S4) and the lowest among reports of carbon-based indoor perovskite solar cells (Table S5). To demonstrate possible usage, 2 Cs10Br30 devices were connected in series to achieve the combined active area of 2 cm^2^, exhibiting an average indoor PCE of 24.74% under LED 1000 lux (Figure 7E); the combined device was able to continuously power a thermo-hygrometer (HTC-2 model) for relative humidity, temperature, and time readings as shown in Figure 7F. The solar cell can provide electrical energy beyond the requirement of the thermo-hygrometer under LED ∼1000 lux as illustrated by the lux meter on the left; the volage readings across the supercapacitor are gradually increasing over time with the operation of the thermo-hygrometer for 30 h, pointing to energy accumulation, as indicated in Figure S17. In addition, the lux meter for 1000 lux measurement was calibrated with a certified spectroradiometer (the certificate was attached in Figure S18). Figure S19 shows the spectrum of the LED light source (4W, 6500 k Phillips) used for our experiment. When the lux meter reads the value of 1000 lux, the real value reading of the light illumination from the certified spectroradiometer is 947 lux with the power density 0.30277 mW cm^−2^. Therefore, with slightly lower light intensity and power density used for our solar performance calculation, the reported solar cell values (PCE, J_sc_, V_oc_) in this work should be slightly underestimated compared to the real values. The light source calibration is demonstrated in Figure S20.

### Conclusions

In summary, the compositional landscape of Cs (5–40%) and Br (17 and 30%) in the triple cation perovskite system was systematically investigated for indoor solar cell application. The absorbance ranges were adjusted via composition tuning to obtain band gaps between 1.64 and 1.77 eV to match the indoor light spectrum. With high Cs, AFM topology measurement reveals wrinkle morphology, leading to a poor interface between perovskite and HTL. Wide-band gap perovskite Cs10Br30 shows high crystallinity along with low microstrain. Furthermore, recombination mechanisms of different compositions were investigated via ideality factor (n_id_) calculation, PL lifetime, and SCLC measurement. Cs10Br30 exhibits comparatively low defects and the highest average indoor PCE of 27.70% with a champion PCE of 31.94% for an active area of 0.04 cm^2^. In addition, light induced phase segregation of wide-band gap Cs10Br30 was clarified by using PL technique. Although phase segregation was found after 1-sun soaking, there is no red shifting after LED light soaking, suggesting promising device for indoor applications. For the stability test, Cs10Br30 retains 90% of initial PCE after 60 days under LED 1000 lux, 8 h/day in ambient air without encapsulation. As solar cell size and HI impact real-world indoor usage, we further optimized both device configuration and dimension to achieve perovskite devices with the ultralow HI of 0.1 and the highest indoor PCE of 30.09% for an active area 1 cm^2^. Our prototype from combining two such devices in series was able to continuously power multiple sensors at the same time.

### Limitations of the study

For PL decay experiment, fluence-dependent measurements for the PL decay could not be performed, as we used a simple benchtop Horiba system.

## STAR★Methods

### Key resources table

**Table:** 

REAGENT or RESOURCE	SOURCE	IDENTIFIER
Lead(II) iodide (PbI_2_)	Tokyo Chemical Industry	L0279
Lead(II) bromide (PbBr_2_)	Tokyo Chemical Industry	L0288
Methylammonium chloride (MACl)	Sigma Aldrich	8.06020
Cesium iodide (CsI)	Sigma Aldrich	202134
4-tert-butylpyridine (tBP),	Sigma Aldrich	142379
Lithium bis-(trifluoromethanesulfonyl) imide (Li-TFSI)	Sigma Aldrich	15224
Copper(I) thiocyanate (CuSCN)	Sigma Aldrich	298212
Formamidinium iodide (FAI)	Greatcell solar	MS150000
Formamidinium bromide (FABr)	Greatcell solar	MS350000
Methylammonium bromide (MABr)	Greatcell solar	MS301000
Tin(IV) oxide, 15wt% in H_2_O colloidal dispersion	Alfa Aesar	44592.36
Ferrocene	Alfa Aesar	087202
Spiro-OMeTAD	Feiming chemical	207739-72-8

### Resource availability

#### Lead contact

Further information and requests for resources should be directed to and will be fulfilled by the lead contact, Associate Professor Pongsakorn Kanjanaboos (Pongsakorn.kan@mahidol.edu).

#### Materials availability

The study did not generate new unique materials. The readers can buy the chemicals to remake the materials as mentioned in the text.

#### Data and code availability

Data: All data reported in this paper will be shared by the lead contact upon request.

Code: This paper does not report the original code.

Any additional information required to reanalyze the data reported in this paper is available from the lead contact upon request.

### Method details

#### Materials

Lead(II) iodide (PbI_2_; 99.99%, trace metals basis) and lead(II) bromide (PbBr_2_; >98.0%) were purchased from Tokyo Chemical Industry (TCI). Methylammonium chloride (MACl; 98%), cesium iodide (CsI; 99.9%, trace metals basis), anhydrous N, N-dimethylformamide (DMF; 99.8% v/v), anhydrous dimethyl sulfoxide (DMSO, 99% v/v), anhydrous chlorobenzene (99.8%), anhydrous ethyl acetate (99.8%), anhydrous ethanol (ethanol; 99.5% v/v), tin(II) chloride dihydrate (SnCl_2_·2H_2_O; 99.999%), hydrochloric acid (HCl; 37% v/v), 4-tert-butylpyridine (tBP), lithium bis-(trifluoromethanesulfonyl) imide (Li-TFSI), acetonitrile (anhydrous; 99.8%), copper(I) thiocyanate (CuSCN; 99%), and diethyl sulfide 98% were purchased from Sigma Aldrich. Propan-2-ol (AR grade) was purchased from RCI labscan. Formamidinium iodide (FAI; >99.99%), formamidinium bromide (FABr; >99.99%), and methylammonium bromide (MABr; >99.99%), and TEC15 FTO glass plate (2.2 mm thick) were purchased from Greatcell solar. TEC15 ITO glass (1.1 mm thick) was purchased from Luminescence Technology Corp. Tin(IV) oxide (15wt% in H_2_O colloidal dispersion) and ferrocene (99%) were purchased from Alfa Aesar. 2,20,7,70-tetrakis[N,N-bis(4-methoxyphenyl)amino]-9,90-spirobifluorene (spiro-OMeTAD) was purchased from Feiming chemical. Alconox detergent powder was purchased from Alconox. Carbon paste (Jelcon CH-8) was purchased from Jujo chemical.

#### Device fabrication

FTO glasses were cleaned by first sonicating in Alconox detergent (10 g in 500 mL of DI water) for 30 min, then rinsed with DI water for 3 times, sonicated in IPA for 30 min, and dried with a nitrogen gun. Cleaned FTO glasses were treated under a UV-ozone cleaner within 10 min before deposition.

For single-layer ETL, 0.2 M of SnCl_2_·2H_2_O solution was prepared by dissolving in anhydrous ethanol. The ETL solution was spin coated on cleaned FTO glass with the spin rate of 3000 rpm for 30 s with the initial acceleration of 1500 rpm/s, followed by annealing at 180°C for 1 h.

For bi-layer ETL, 0.1 M of SnCl_2_·2H_2_O solution was spin coated on FTO with the spin rate of 5000 rpm for 30 s with the initial acceleration of 2500 rpm/s, then annealed at 180°C for 1 h. The second layer of SnO_2_ was prepared by diluting 15 wt% SnO_2_ dispersion with DI water in the ratio 1:2; the solution was then spin coated on the first ETL layer with the spin rate of 5000 rpm for 30 s with the initial acceleration of 2500 rpm s^−^^1^ and then annealed at 150°C for 30 min.

For perovskite precursor solution, 1.25 M of PbI_2_ and PbBr_2_ solutions in DMF:DMSO (4:1) were separately prepared. FAI and CsI powders were added into two separate bottles of PbI_2_ solution to obtain 1.14 M FAPbI_3_ and CsPbI_3_ solutions, respectively. Similarly, FABr and MABr powders were added into separate bottles of the PbBr_2_ solution to obtain 1.14 M FAPbBr_3_ and MAPbBr_3_ solutions, respectively. The final mixture was prepared by mixing of FAPbI_3_, FAPbBr_3_, MAPbBr_3_, and CsPbI_3_ solutions, which were separately prepared in the previous steps, together in the stoichiometric ratios to achieve desired triple cation perovskite Cs_x_(FA_0.88_MA_0.12_)_1-x_Pb(I_1-y_Br_y_)_3_ where x = 0.05, 0.10, 0.20, 0.30, and 0.40 while y = 0.17 or 0.30. The 0.4 M of excess MACl in DMF:DMSO (4:1) was added to the triple cation perovskite solution with 5% volume ratio. The mixed solution was stirred at room temperature for 1-2 h and was filtered with a 0.2 μm PTFE filter before use.

The finished perovskite solution (60 μL) was spin coated on a FTO/SnO_2_ substrate in a 2-step process: 1) the spin speed of 1000 rpm for 10 s with the initial acceleration of 500 rpm s^−^^1^ and 2) the spin speed of 4000 rpm for 30 s with the initial acceleration of 2000 rpm s^−^^1^. Ethyl acetate (130 μL) as an anti-solvent was dropped at the 30^th^ s (counted from the beginning of the spin coating process); the film was then annealed at 100°C for 30 min under N_2_ filled glove box.

For spiro-OMeTAD HTL, 10 mg/mL of ferrocene in chlorobenzene was spin coated on FTO/SnO_2_/Perovskite with the spin speed of 4000 rpm for 20 s with the initial acceleration of 2000 rpm s^−^^1^. 80 mg of spiro-OMeTAD was dissolved in 1 mL chlorobenzene. Then 17.5 μL of Li-TFSI solution (520 mg in 1 mL acetonitrile) and 28.5 μL of 4-tertbutyl pyridine were added into the spiro-OMeTAD solution as additives and then stirred overnight at room temperature in the dark. The solution (50 μL) was spin coated on a perovskite film at the speed of 2000 rpm for 30 s with the initial acceleration of 1000 rpm s^−^^1^ under N_2_ filled glove box.

For CuSCN HTL, 35 mg of CuSCN was dissolved in 1 mL of diethyl sulfide for 5 h and filtered with a 0.2 μm PTFE filter. FTO/SnO_2_/Perovskite was first set to spin at 4000 rpm; while spinning, the solution (50 μL) was dropped and the spin process then continued for another 25 s.

A carbon electrode sheet was prepared by doctor blading the commercial carbon paste over a 120-μm deep well to control electrode thickness and the wet carbon was then soaked in ethanol for 2 h. The carbon sheet was then peeled off the well and left to dry before use. The carbon sheet (∼120 μm thick) was cut into the required size (0.04 cm^2^ or 1 cm^2^) and aligned on FTO/ETL/Perovskite/HTL. Lastly, a cleaned ITO substrate was placed on top and the whole stack was hot pressed at 0.6 MPa and 60°C for 5 min. The finished stack was pressed again at 0.6 MPa for 5 min at room temperature. All carbon electrode assembly was done under ambient air.

#### Device characterization

Absorbance spectra were measured by Shimadzu UV-2600 UV-Vis spectrophotometer with the scanning range from 350 to 900 nm. Photoluminescence data was determined via a Horiba FluoroMax-plus spectrophotometer (excitation wavelength of 470 nm, 0.3 s integration time, and bandpass slit size of 3 nm). Photoluminescence lifetime was recorded by Horiba FluoroMax-plus coupled with Horiba DeltaHub system (excitation: 635 nm NanoLED, detection: bandpass of 29.4 nm, 675 nm high performance longpass filter (OD 4.0)) recorded from the substrate side probed at 740, 710, and 705 nm for Cs10Br17, Cs10Br30, and Cs20Br30, respectively. Ultraviolet photoelectron spectroscopy (UPS) was measured at beamline 3.2 Ua at Synchrotron Light Research Institute, Thailand, using photon energy of 60 eV. Film crystallinity was characterized by D8 Discover Bruker X-ray diffractometer (x-ray source Cu Kα, scanning step size of 2θ of 0.01°, time/step of 0.4 s). Perovskite surfaces and cross-sectional images were taken with field emission scanning electron microscope (FE-SEM, Joel, JSM-7610Plus, and acceleration voltage of 7 kV). Topography images and current maps were done by conductive atomic force microscopy (Park NX-10, room ambient, ANSCMPC conductive contact probe with a spring constant of k = 0.036 N m^−^^1^ and resonant frequency of 15 kHz, a scan speed of 1.25 μm/s, and 2 nN set point). Under indoor white light illumination at 285.75 mW cm^-2^, the short circuit current (I_sc_) map was done at 0 V, while the open circuit voltage (V_oc_) map was done at 0.5 V in the forward direction. Elemental investigation was performed by X-ray fluorescence microscope (Horiba, XGT-9000, voltage of 50 kV, current of 1 mA, and spot diameter of 100 μm). Indoor solar cell performances were measured under 1000 lux (0.31 mW cm^-2^, 6500 K Phillip E27 LED 4 W, scan step of 0.05 V, and a scan range from -0.1 V to 1.1 V, scan rate of 200 mV s^−^^1^). 1-sun performances were measured under AAA-class 7520-LED light source with LSS-7120 LED controller (VeraSol). The 100 mW cm^-2^ of 1-sun illumination was calibrated by Si diode (Hamamatsu S1133). J-V measurement of under 1-sun was scanned by using 0.01 V step size and voltage sweep from -0.1 V to 1.2 V (scan rate 47 mV s^−^^1^). Both indoor and 1-sun measurements were achieved via Keithley 2400 source. EQE spectra were measured using Enlitech QE-R quantum efficiency analyzer (DC mode with 0.04 mm^2^ beam diameter).

## Supplemental information

Document S1. Figures S1–S20 and Tables S1–S5

Data S1. Standardized data reporting for photovoltaic cells
