# Supplementary material for: Cs and Br tuning to achieve ultralow-hysteresis and high-performance indoor triple cation perovskite solar cell with low-cost carbon-based electrode
Source: iScience. 2024 Feb 22;27(4):109306. doi: 10.1016/j.isci.2024.109306 (PMC10940937; doi:10.1016/j.isci.2024.109306)
Supplement: Document S1. Figures S1–S20 and Tables S1–S5 [file mmc1.pdf]

iScience, Volume *27*

Supplemental information

Cs and Br tuning to achieve ultralow-hysteresis and high-performance indoor triple cation perovskite solar cell with low-cost carbon-based electrode

Ladda Srathongsian, Anusit Kaewprajak, Atittaya Naikaew, Chaowaphat Seriwattanachai, Napan Phuphathanaphong, Anuchytt Inna, Thana Chotchuangchutchaval, Woraprom Passatorntaschakorn, Pisist Kumnorkaew, Somboon Sahasithiwat, Duangmanee Wongratanaphisan, Pipat Ruankham, Ratchadaporn Supruangnet, Hideki Nakajima, Pasit Pakawatpanurut, and Pongsakorn Kanjanaboos

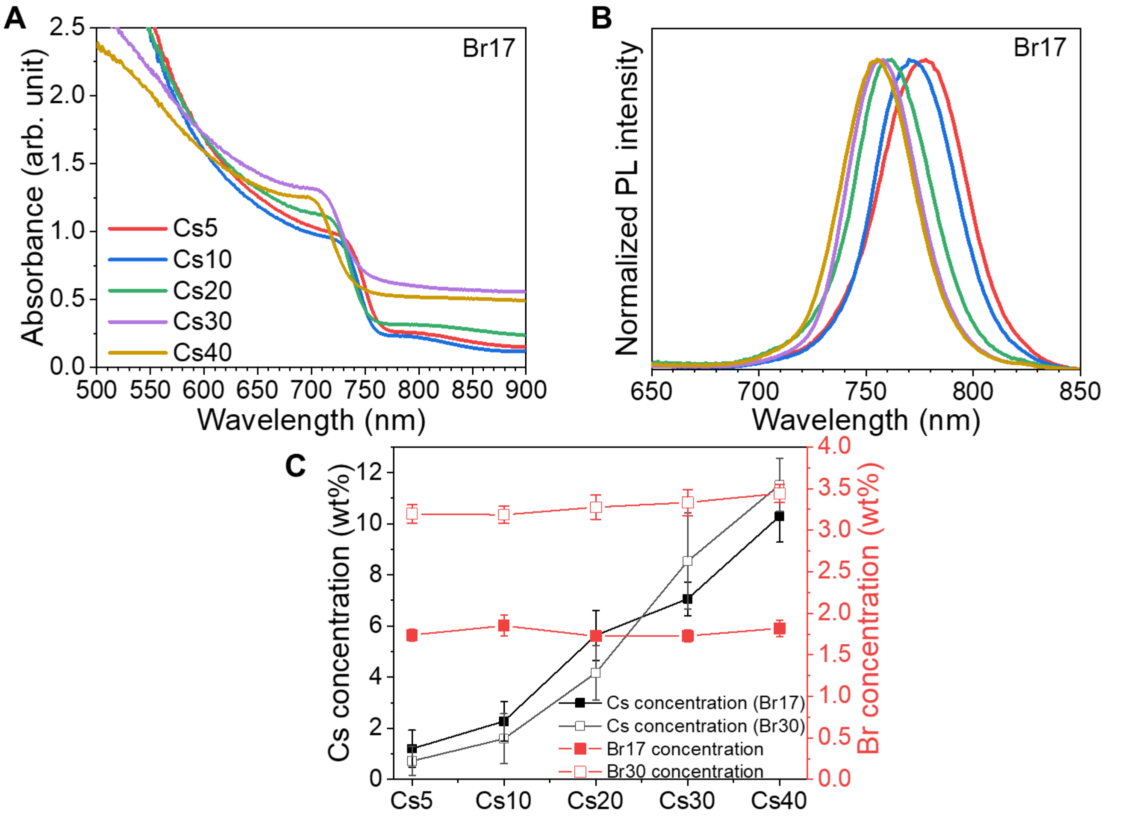

Figure S1. Composition analysis, related to Figure 1.

(A) Absorbance of Br17 with 5-40%. (B) PL spectra of Br17 with 5-40% Cs. (C) Cs and Br composition plots from XRF measurement.

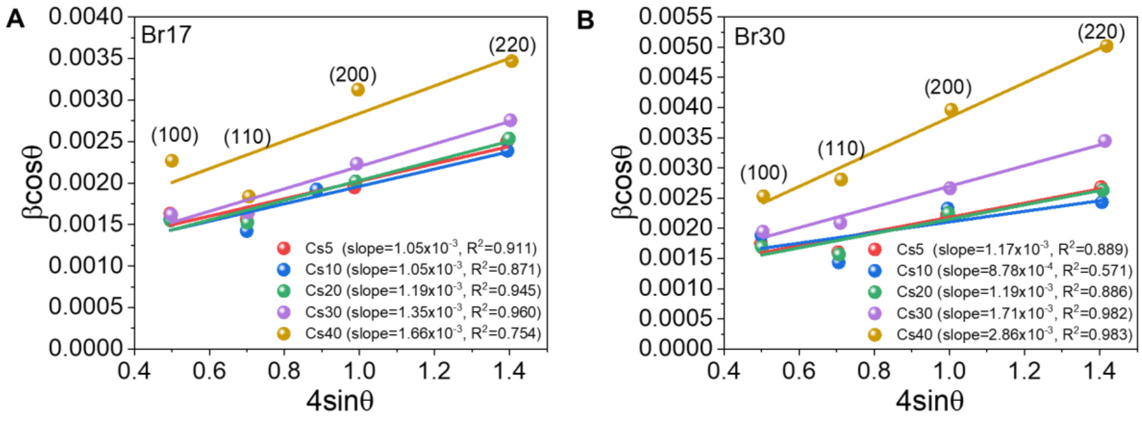

Figure S2. Williamson-Hall plots, related to Figure 2.

Williamson-Hall plots of (A) Br17 and (B) Br30.

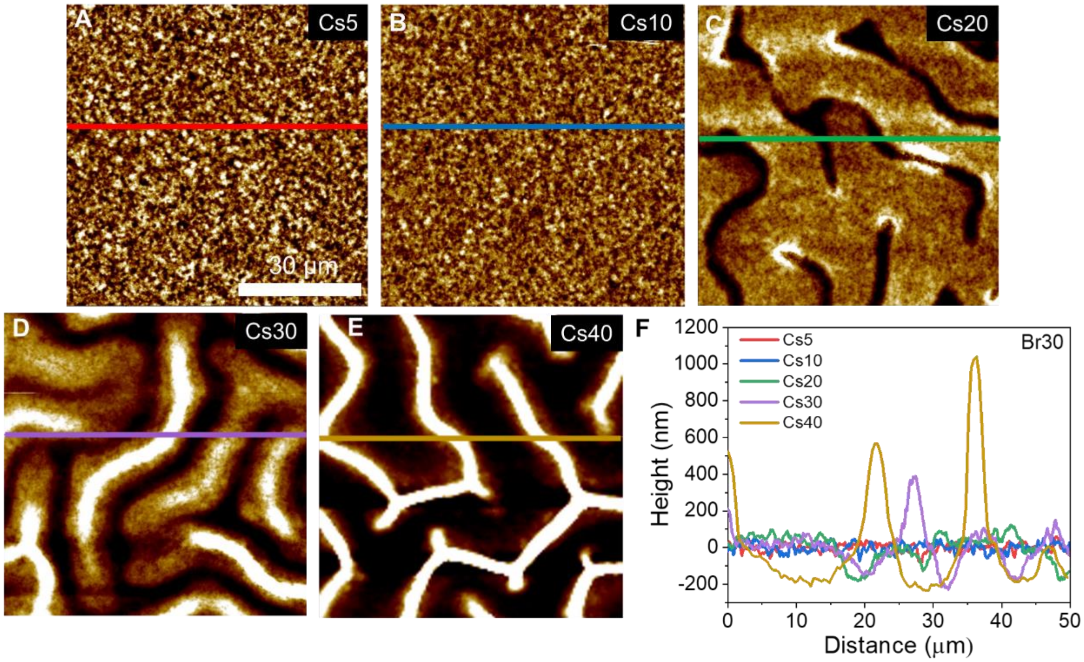

Figure S3. AFM images and surface line profile, related to Figure 3.

(A-E) AFM topography images of Br30 perovskite films with Cs5, Cs10, Cs20, Cs30, and Cs40, respectively. (F) Line profile of Br30 perovskite films with different Cs contents.

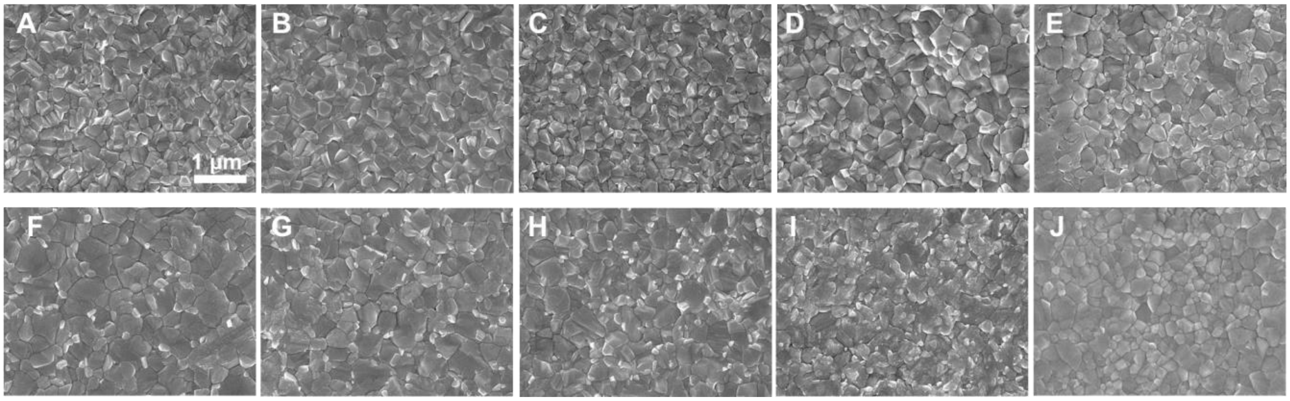

Figure S4. SEM images, related to Figure 3.

(A-E) SEM images of Br17 perovskite with Cs5, Cs10, Cs20, Cs30, and Cs40, respectively. (F-J) SEM images of Br30 perovskite with Cs5, Cs10, Cs20, Cs30, and Cs40, respectively.

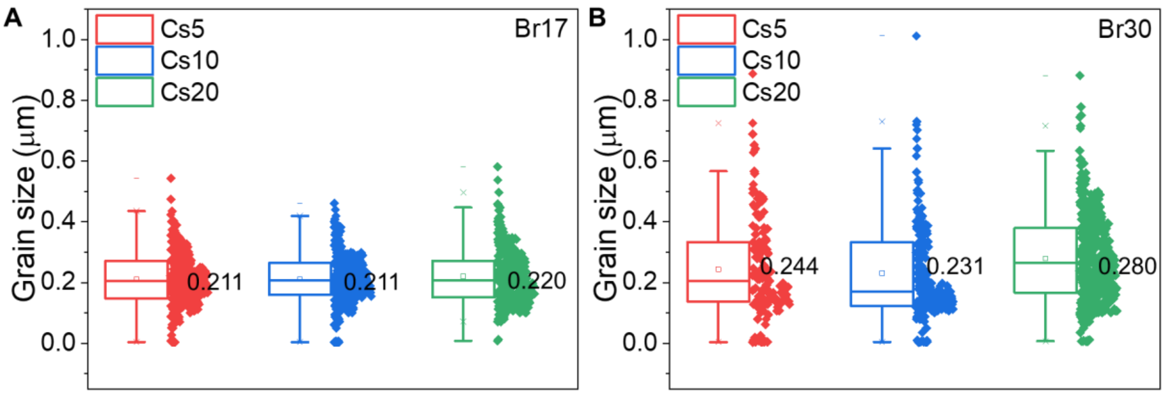

Figure S5. Grain size analysis, related to Figure 3.

(A) Grain size distribution boxplots of Br17 perovskite films with Cs5, Cs10, and Cs20. (B) Grain size distribution boxplots of Br30 perovskite films with Cs5, Cs10, and Cs20.

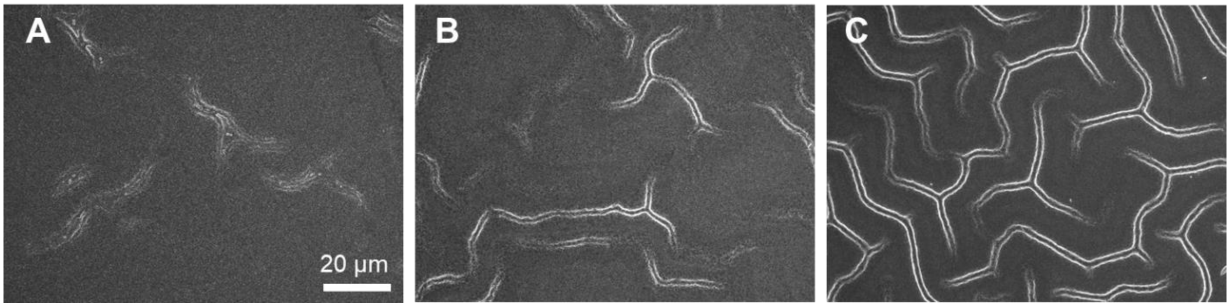

Figure S6. SEM images, related to Figure 3.

SEM images of Br30 perovskite films with (A) Cs20, (B) Cs30, and (C) Cs40.

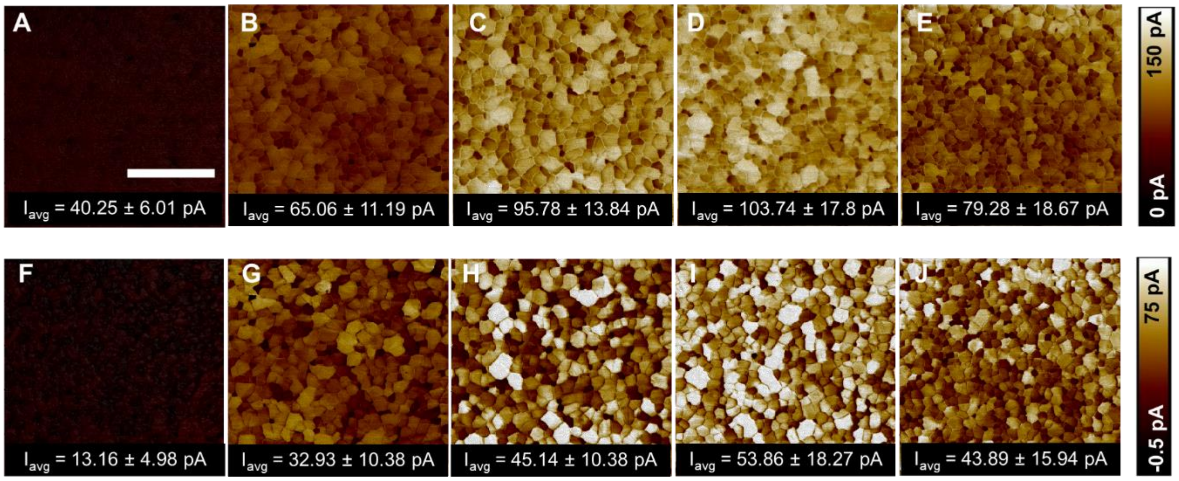

Figure S7. AFM current mapping, related to Figure 3.

Current mapping at 0 V bias of Br30 perovskite films with (A) Cs5, (B) Cs10, (C) Cs20, (D) Cs30, and (E) Cs40. Current mapping at 0.5 V (the V_{oc} condition) of Br30 perovskite films with (F) Cs5, (G) Cs10, (H) Cs20, (I) Cs30, and (J) Cs40.

The high current at the grain boundaries (GBs) was observed for all of our samples at 0 V bias under white light source (285.75 mW cm^{-2}). The high photocurrent at the GBs was previously investigated by Li *et al*. In the work, an external bias was intentionally applied to enhance charge transportation (the bias direction was in the same direction with the self-generated E-field of the $MAPbI_3$ film) to overcome charge barrier at the GBs; current mapping with 0.3 V bias shows high photocurrent at GBs than that of 0 V bias[1]. However, our work with triple cation perovskite films on ETL shows high photocurrent at GBs with 0 V bias, meaning that only the bias from self-generated E-field of our perovskite film is sufficient to break the charge barrier at the GBs. Another work from Li *et al.* demonstrated that perovskite films with and without DMF treatment show different charge carrier break down bias voltages for generating high photocurrent at the GBs.[2]

Moreover, in this work, when external bias was given in the opposite direction of the self-generated bias from our perovskite films on ETL (our V_{oc} condition in Figure S7 F-J), the absence of high photocurrent at GBs was observed in agreement with above discussion.

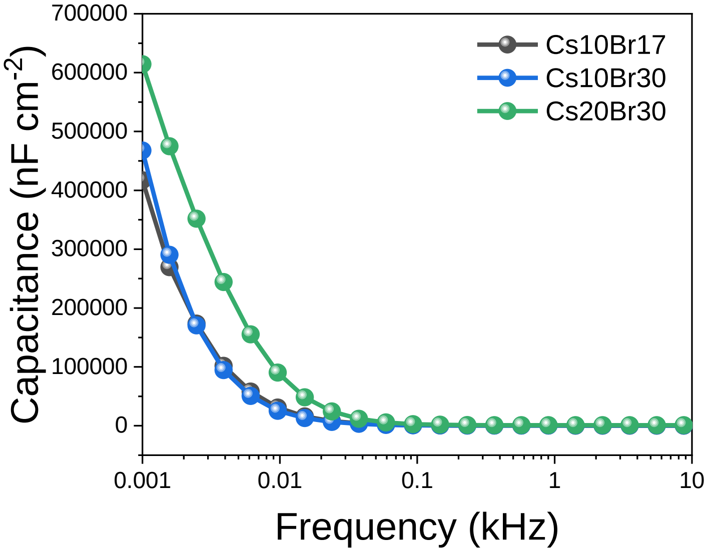

Figure S8. Capacitance measurement of perovskite solar cell devices, related to Figure 4.

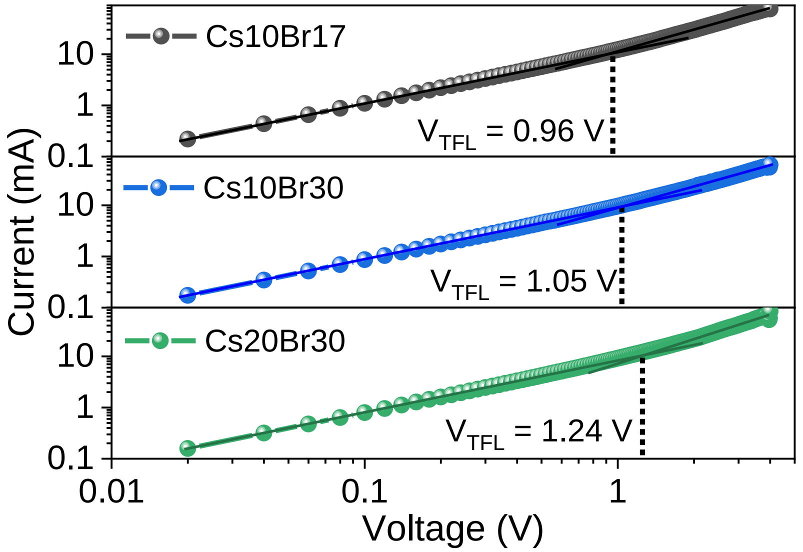

Figure S9. Space-charge-limited current (SCLC) characterization with electron-only devices (FTO/SnO_2/Perovskite/PCBM/Carbon/ITO) where perovskite is Cs10Br17, Cs10Br30, or Cs20Br30, related to Figure 4.

Trap density is calculated by following equation; $N_{trap} = 2\varepsilon\varepsilon_0 V_{TFL}/qd^2$, N_{trap} is linearly proportional to V_{TFL}; however, the density is inversely proportional to the thickness (d^2). Although V_{TFL} value increases with higher Cs concentration, the thicker film from high Cs concentration (from wrinkle features) reduces N_{trap} value, resulting in the same trap density for the three compositions. Furthermore, for the trap calculation, we used weighted average thicknesses by taking both valleys and hills due to the wrinkle areas into account. However, the trap density equation is meant for a relatively flat film; hence, our calculated trap density values may not be fully reliable.

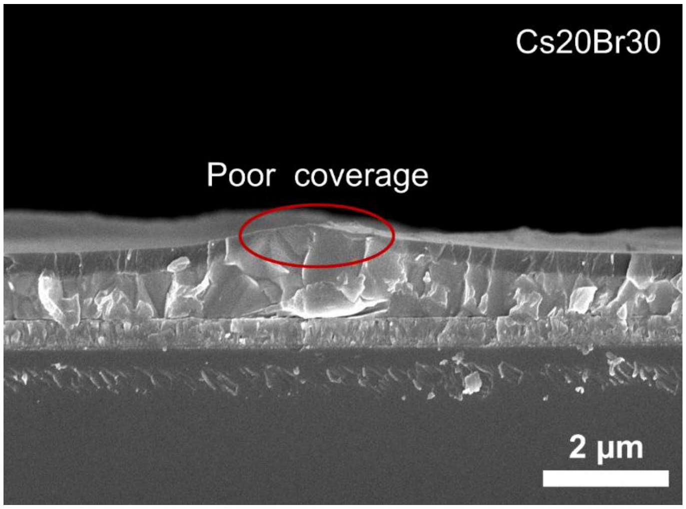

Figure S10. Cross-sectional SEM image of FTO/SnO_2/Cs20Br30/Spiro-OMeTAD, related to Figure 5.

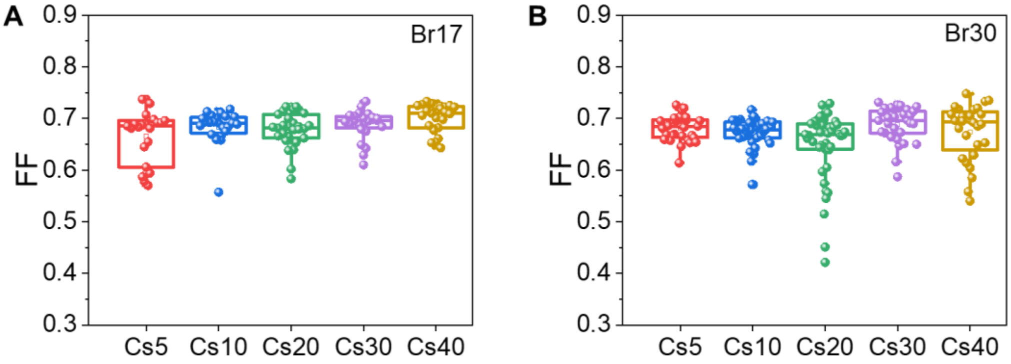

Figure S11. Boxplot of FF under indoor light 1000 lux, related to Figure 5.

(A) Br17, and (B) Br30.

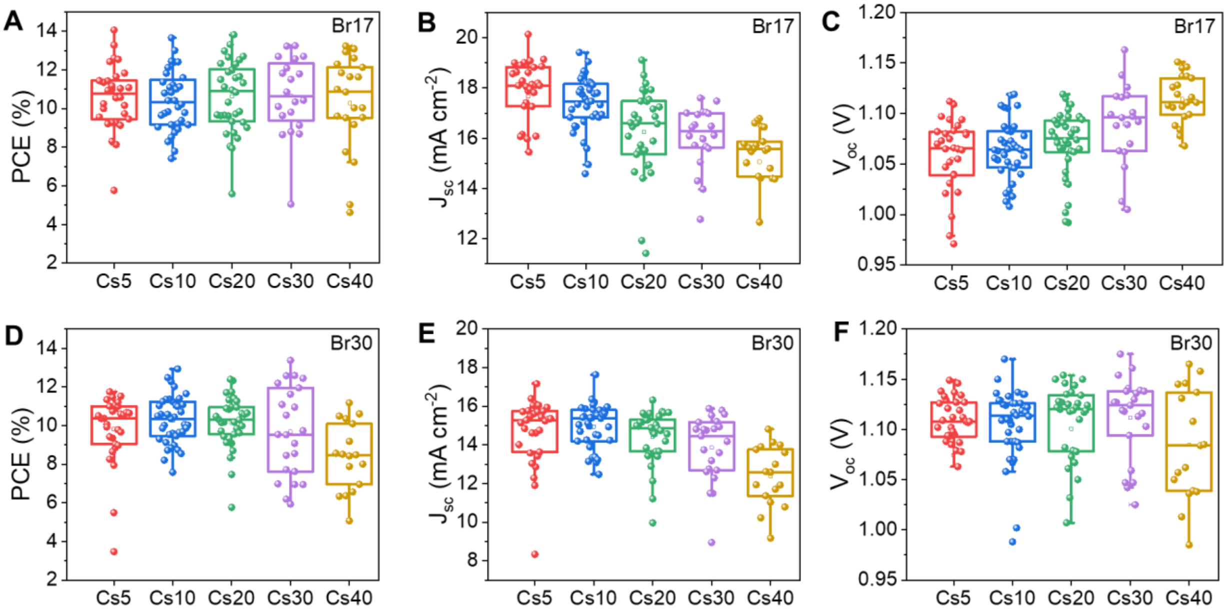

Figure S12. Device performance parameters under 1-sun, related to Figure 5.

(A) PCE, (B) J_{sc}, (C) and V_{oc} for Br17; (D) PCE, (E) J_{sc}, and (F) V_{oc} for Br30.

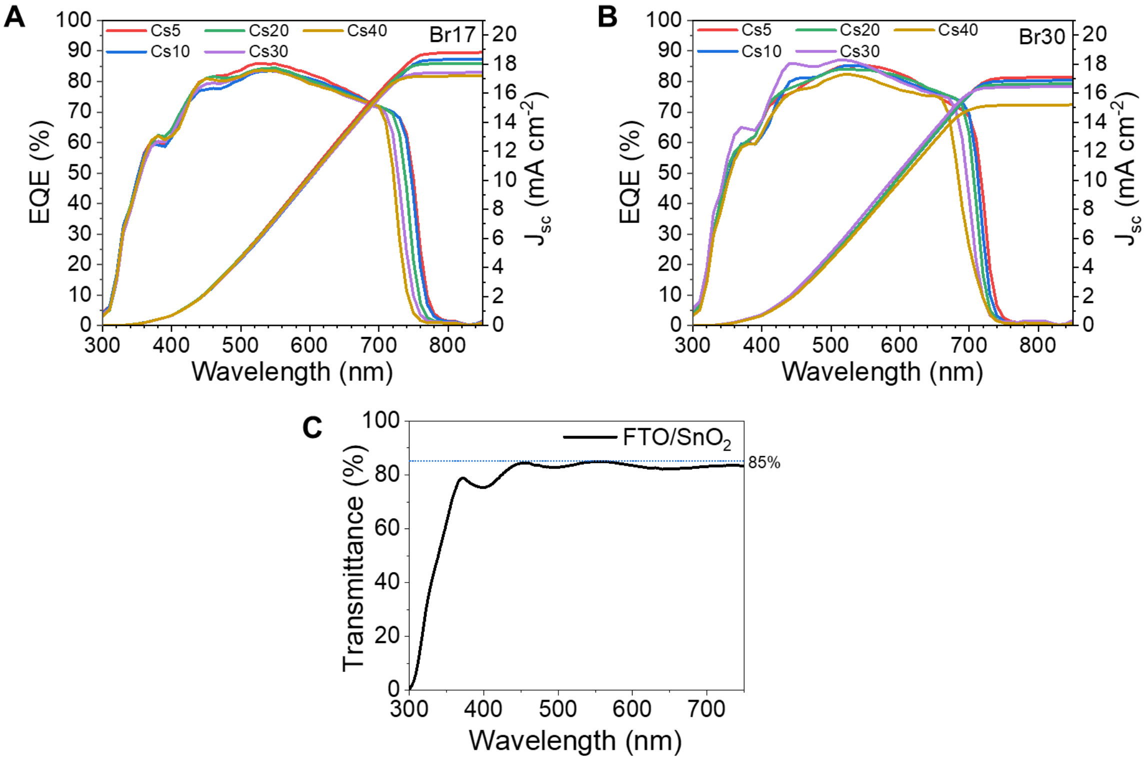

Figure S13. EQE and transmittance of substrate, related to Figure 5.

(A) EQE of Br17 perovskite devices. (B) EQE of Br30 perovskite devices. (C) Transmittance of FTO/SnO_2.

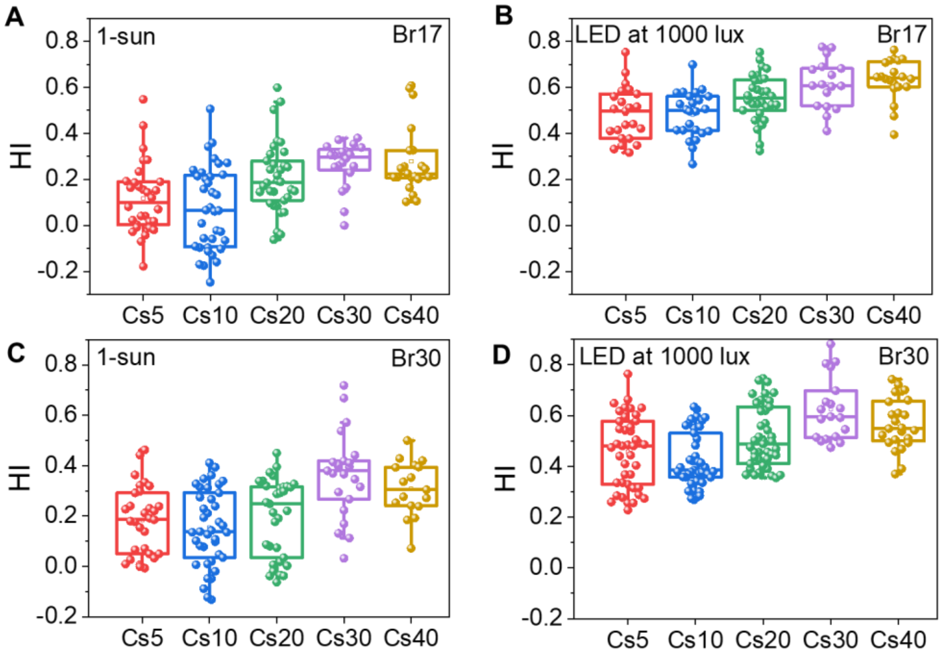

Figure S14. HI, related to Figure 5.

(A) under 1-sun, (B) under LED at 1000 lux for Br17, (C) under 1-sun, and (D) under LED at 1000 lux for Br30.

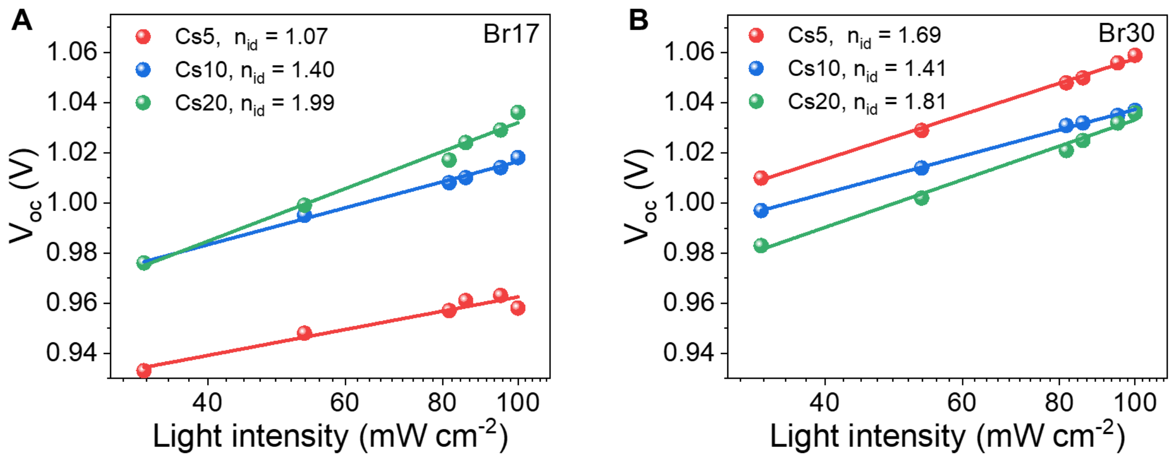

Figure S15. Ideality factor analysis, related to Figure 4.

V_{oc}-light intensity dependence of (A) Br17 devices with Cs5, Cs10, and Cs20 and (B) Br30 devices with Cs5, Cs10, and Cs20.

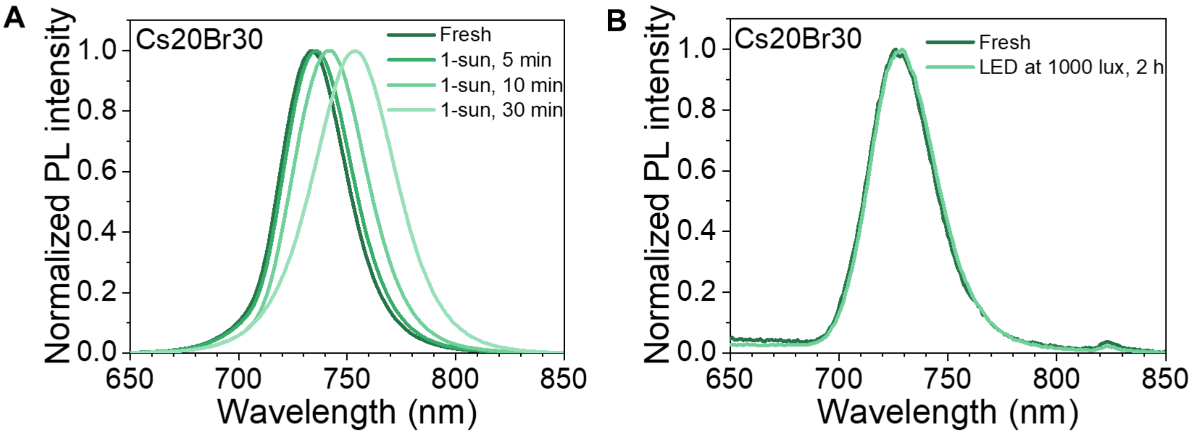

Figure S16. Photostability of Cs20, related to Figure 6.

Normalized PL spectra of Cs20Br30 after (A) Soaking in 1-sun for 5–30 min and (B) Soaking in LED at 1000 lux for 2 h.

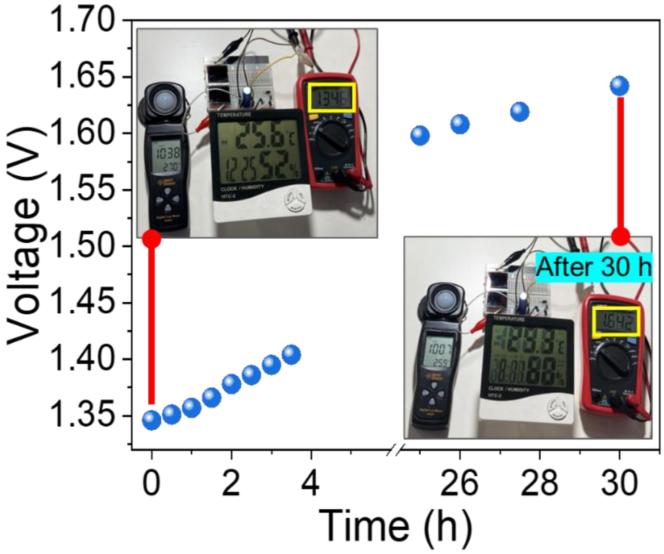

Figure S17. Voltage reading of supercapacitor powered by indoor perovskite solar cell over time with non-stop operation of thermo-hygrometer (HTC-2 model), related to Figure 7.

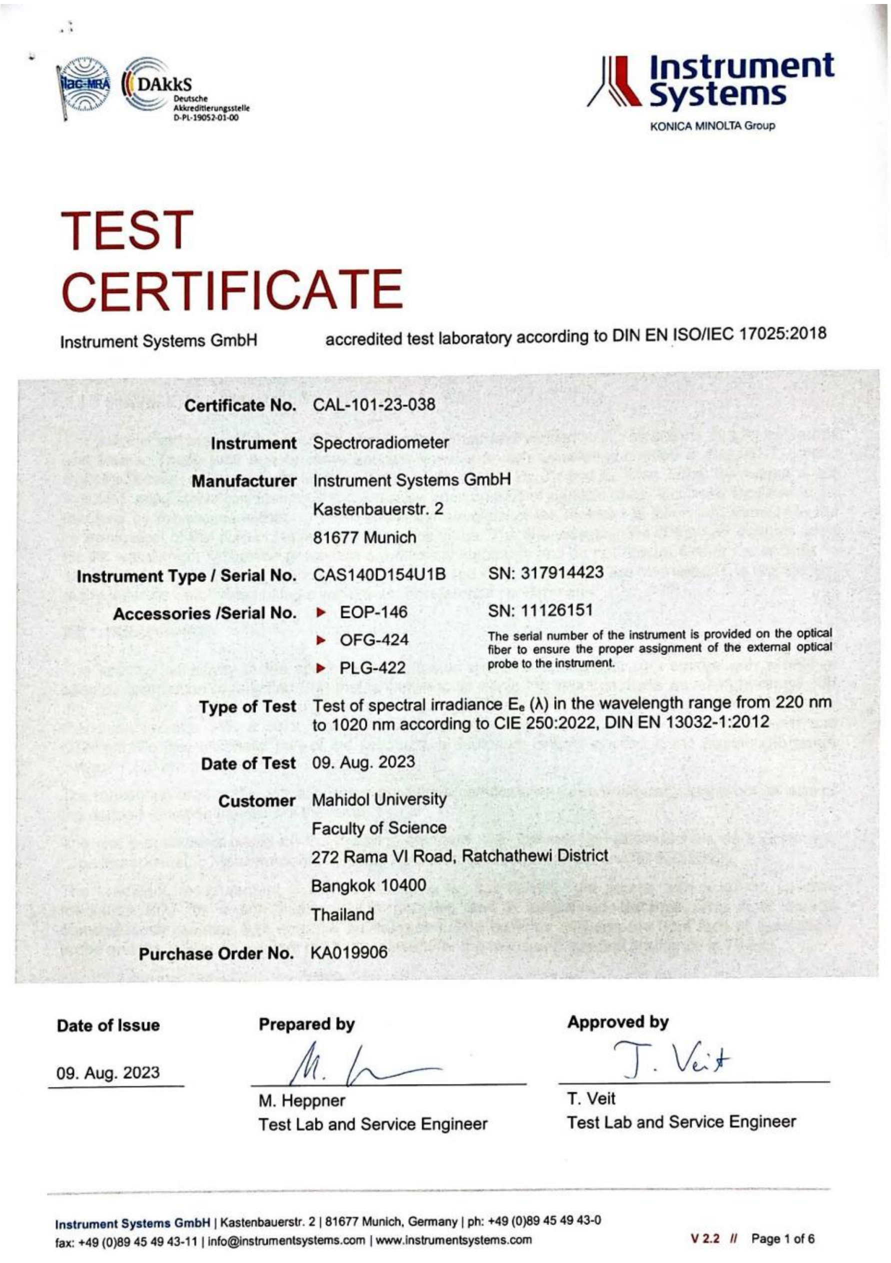

Instrument Systems
KONICA MINOLTA Group

DAkkS Deutsche Akkreditierungsstelle D-PL-19052-01-00

TEST CERTIFICATE

Instrument Systems GmbH accredited test laboratory according to DIN EN ISO/IEC 17025:2018

**Certificate No.**	CAL-101-23-038
**Instrument**	Spectroradiometer
**Manufacturer**	Instrument Systems GmbH<br>Kastenbauerstr. 2<br>81677 Munich
**Instrument Type / Serial No.**	CAS140D154U1B SN: 317914423
**Accessories /Serial No.**	▸ EOP-146 SN: 11126151<br>▸ OFG-424<br>▸ PLG-422<br>The serial number of the instrument is provided on the optical fiber to ensure the proper assignment of the external optical probe to the instrument.
**Type of Test**	Test of spectral irradiance E_e (λ) in the wavelength range from 220 nm to 1020 nm according to CIE 250:2022, DIN EN 13032-1:2012
**Date of Test**	09. Aug. 2023
**Customer**	Mahidol University<br>Faculty of Science<br>272 Rama VI Road, Ratchathewi District<br>Bangkok 10400<br>Thailand
**Purchase Order No.**	KA019906

**Date of Issue**	**Prepared by**	**Approved by**
09. Aug. 2023	M. Heppner<br>Test Lab and Service Engineer	T. Veit<br>Test Lab and Service Engineer

Instrument Systems GmbH | Kastenbauerstr. 2 | 81677 Munich, Germany | ph: +49 (0)89 45 49 43-0
fax: +49 (0)89 45 49 43-11 | info@instrumentsystems.com | www.instrumentsystems.com V 2.2 // Page 1 of 6

Figure S18. Test certificate of spectroradiometer, related to Figure 7.

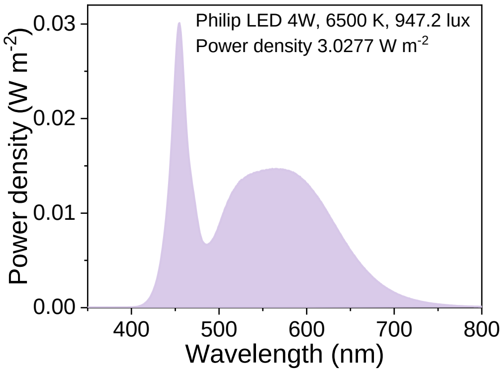

Figure S19. LED spectrum measured by a certified spectroradiometer (Instrument Systems with EOP-146), related to Figure 7.

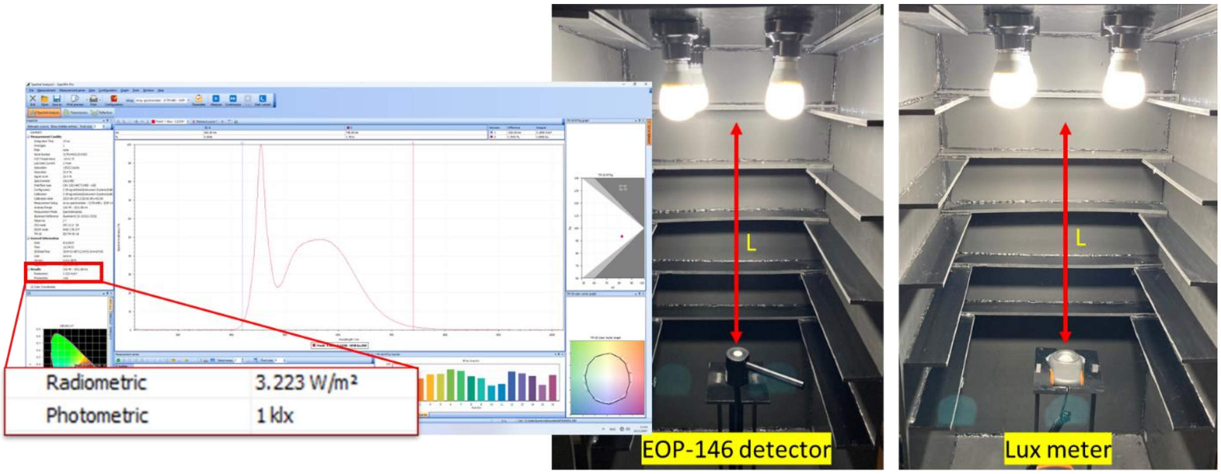

Figure S20. Indoor light source spectrum measurement and the lux meter calibration, related to Figure 7.

The indoor light source spectrum of the testing box (6500 K LED) was measured directly by spectroradiometer with a certified EOP-146 detector. The position of spectroradiometer detector was set to a position with the illumination of 1000 lux. The EOP-146 can provide a certified power density along with the spectrum information. Therefore, the obtained spectrum and the power density are accurate.

Additionally, we calibrated our lux meter with the same certified spectroradiometer at the same height. So, the lux meter can be later used to do height adjustment during solar cell testing along with the certified spectroradiometer.

Table S1. PL lifetime fitting parameters by using the bi-exponential decay equation. The average lifetime is obtained from $\tau_{avg} = A_1 \tau_1 + A_2 \tau_2$, related to Figure 4.

Sample (on glass)	A_1 [%]	τ_1 [ns]	A_2 [%]	τ_2 [ns]	τ_{avg} [ns]	χ^2
Cs10Br17	61	460	39	1639	919	1.10
Cs10Br30	71	377	29	2007	854	1.15
Cs20Br30	72	345	28	1651	711	1.16

Table S2. Perovskite film thickness measurement from SEM images, related to Figure 4.

Sample	Film thickness (nm)
Cs10Br17	561
Cs10Br30	602
Cs20Br30	645

Table S3. Indoor performance parameters (reverse scan) under LED at 1000 lux with an active area of 1 cm^2 and device configuration FTO/sol-gel SnO_2/SnO_2 NPs/Perovskite/CuSCN/Carbon/ ITO, related to Figure 7.

Device	PCE_{RS} [%]	J_{sc} [μA cm^{-2}]	V_{oc} [V]	FF	HI[a)]
1	26.12	116	0.940	0.746	0.073
2	27.04	120	0.945	0.742	0.116
3	26.98	116	0.944	0.762	0.149
4	30.09	124	0.955	0.790	0.123
5	23.10	105	0.924	0.737	0.068
6	22.57	109	0.935	0.687	0.038
7	25.25	111	0.929	0.760	0.033
8	24.12	118	0.857	0.738	0.203
9	23.31	116	0.864	0.720	0.178
10	23.50	108	0.917	0.734	0.067
11	26.74	113	0.932	0.787	0.130
12	24.50	112	0.911	0.741	0.073
13	22.80	116	0.860	0.709	0.101
14	24.71	114	0.921	0.731	0.173
15	22.42	108	0.913	0.708	0.109
16	21.31	108	0.836	0.688	0.082
Average	24.66	113	0.911	0.736	0.107
SD	2.270	5.097	0.037	0.030	0.050

HI [a)] = (PCE_{RS}-PCE_{FS})/PCE_{RS}

Table S4. **Summary of metal-based indoor perovskite solar cell performances (n-i-p structure), related to Figure 7.**

Device configuration	Band gap [eV]	Light source	J_{sc} [μA cm^{-2}]	V_{oc} [V]	FF [%]	PCE [%]	HI	Ref.
ITO/SnO_2/MgO/$MAPbI_3$/ Spiro-MeOTAD/Au	NA	400 lux LED,4 W daylight	64.5	0.895	72.0	26.9	0.119	3
ITO/SnO_2/$MAPbI_3$/ Spiro-OMeTAD/Au	1.57	1000 lux LED	NA	0.86	75.3	27.0	8x10^{-4}	4
FTO/TiO_2/$MAPbI_{3-x}Br_x$/ Spiro-OMeTAD/Au	NA	1000 lux LED	170.8	0.821	68.8	34.5	NA	5
FTO/SnO_2/FAMAPbIBrCl/ Spiro-OMeTAD/Au	NA	800 lux LED	98.0	0.84	65	19.2	NA	6
FTO/$Nb_yTi_{1-y}O_2$/ $Cs_{0.05}MA_{0.95}PbBr_xI_{3-x}$/ Spiro-OMeTAD/Au	NA	8.7 W m^{-2} LED	403	0.999	80	36.32	0.030	7
ITO/SnO_2/ZnO/$MAPbI_3$/ Spiro-OMeTAD/Au	NA	1000 lux LED, 6500 K	157.6	0.98	72	35.9	NA	8
FTO/TiO_2/$CsPbBrI_2$/PTAA/Mo O_3/Ag	1.89	1000 lux FL	170	0.75	62	28.48	0.029	9
FTO/TiO_2 /$(FAPbI_3)_{0.92}(MAPbBr_3)_{0.08}$/ CH_3O-PEABr /Spiro-OMeTAD/Au	1.58	824.5 lux LED, 2700 K	152.10	1.001	79.52	40.10	NA	10
FTO/SnO_2/ $Cs_{0.05}(FA_{0.85}MA_{0.15})_{0.95}Pb(I_{0.85}Br_{0.15})_3$/Spiro-OMeTAD/ MoO_3/Ag	NA	1000 lux	156	0.96	79	37.9	NA	11
ITO/SnO_2/SnO_X/$CsPbI_2Br$/ PDTDT/Au	NA	1000 lux LED	96.50	1.23	82.07	32.6	0.050	12
ITO/SnO_2/$MA_{0.9}FA_{0.1}PbI_3$/ Spiro-OMeTAD/Au	1.586	1000 lux LED	186.52	0.91	74.16	34.07	0.070	13
FTO/TiO_2/mp-TiO_2/ $Cs_{0.05}FA_{0.79}MA_{0.16}Pb(I_{0.50}Br_{0.50})$ /Spiro-OMeTAD/Au	NA	200 lux FL	31.02	0.815	78.0	30.3	0.440	14

Table S5. Summary of carbon-based indoor perovskite solar cell performances, related to Figure 7.

Device configuration	Band gap [eV]	Light source	J_{sc} [μA cm^{-2}]	V_{oc} [V]	FF [%]	PCE [%]	Active area [cm^2]	HI	Ref.
FTO/ZTO/ $Cs_{0.17}FA_{0.83}Pb(I_{0.83}Br_{0.17})_3$/CuSCN:CuPc/ Carbon	NA	1000 lux LED, 6500 K	213	0.857	55.6	32.10	0.0385	NA	[15]
FTO/SnO_2/$FA_{0.45}MA_{0.49}Cs_{0.06}Pb(I_{0.62}Br_{0.32}Cl_{0.06})_3$/Spiro-OMeTAD/ Carbon	1.80	1000 lux LED, 6500 K	170	0.93	63	32.0	0.04	0.12	[16]
FTO/c-TiO_2/mp-TiO_2 /$CH_3NH_3PbI_{3-x}Cl_x$/C	NA	200-250 lux	37.65	0.9322	59.55	20.90	88	NA	[17]
FTO/TiO_2-NPs/ $Cs_{0.17}FA_{0.83}Pb(I_{0.83}Br_{0.17})_3$/Spiro-OMeTAD/ Carbon(C-rCP)	NA	1000 lux LED, 6500 K	147.86	0.882	72.43	27.71	1.00	0.133	[18]
FTO/SnO_2/ $Cs_{0.10}(FA_{0.88}MA_{0.12})_{0.90}Pb(I_{0.70}Br_{0.30})_3$/ Spiro-OMeTAD /Carbon	1.73	1000 lux LED, 6500 K	157	0.916	68.4	31.94	0.04	0.414	This work [a)]
FTO/SnO_2/SnO_2 NPs/ $Cs_{0.10}(FA_{0.88}MA_{0.12})_{0.90}Pb(I_{0.70}Br_{0.30})_3$/CuSCN/ Carbon	1.73	1000 lux LED, 6500 K	124	0.955	79.0	30.09	1	0.123	This work [a)]
			113	0.911	73.6	24.66	1	0.107	This work [b)]

[a)]Champion device, [b)] Average value

References

1. Li, J.J., Ma, J.Y., Ge, Q.Q., Hu, J.S., Wang, D., and Wan, L.J. (2015). Microscopic Investigation of Grain Boundaries in Organolead Halide Perovskite Solar Cells. ACS Appl. Mater. Interfaces *7*, 28518–28523. 10.1021/acsami.5b09801.

2. Li, J.J., Ma, J.Y., Hu, J.S., Wang, D., and Wan, L.J. (2016). Influence of N,N-Dimethylformamide Annealing on the Local Electrical Properties of Organometal Halide Perovskite Solar Cells: An Atomic Force Microscopy Investigation. ACS Appl. Mater. Interfaces *8*, 26002–26007. 10.1021/acsami.6b07647.

3. Dagar, J., Castro-Hermosa, S., Lucarelli, G., Cacialli, F., and Brown, T.M. (2018). Highly efficient perovskite solar cells for light harvesting under indoor illumination via solution processed SnO2/MgO composite electron transport layers. Nano Energy *49*, 290–299. 10.1016/j.nanoen.2018.04.027.

4. Cheng, R., Chung, C.C., Zhang, H., Zhou, Z., Zhai, P., Huang, Y.T., Lee, H., and Feng, S.P. (2019). An Air Knife–Assisted Recrystallization Method for Ambient-Process Planar Perovskite Solar Cells and Its Dim-Light Harvesting. Small *15*, 1804465. 10.1002/smll.201804465.

5. Lim, J.W., Kwon, H., Kim, S.H., You, Y.J., Goo, J.S., Ko, D.H., Lee, H.J., Kim, D., Chung, I., Kim, T.G., et al. (2020). Unprecedentedly high indoor performance (efficiency > 34 %) of perovskite photovoltaics with controlled bromine doping. Nano Energy *75*, 104984. 10.1016/j.nanoen.2020.104984.

6. Kim, J., Jang, J.H., Choi, E., Shin, S.J., Kim, J.-H., Jeon, G.G., Lee, M., Seidel, J., Kim, J.H., Yun, J.S., et al. (2020). Chlorine Incorporation in Perovskite Solar Cells for Indoor Light Applications. Cell Reports Phys. Sci. *1*, 100273. 10.1016/j.xcrp.2020.100273.

7. Sun, H., Deng, K., Jiang, Y., Ni, J., Xiong, J., and Li, L. (2020). Realizing Stable Artificial Photon Energy Harvesting Based on Perovskite Solar Cells for Diverse Applications. Small *16*, 1906681. 10.1002/smll.201906681.

8. Noh, Y.W., Jin, I.S., Kim, K.S., Park, S.H., and Jung, J.W. (2020). Reduced energy loss in SnO2/ZnO bilayer electron transport layer-based perovskite solar cells for achieving high efficiencies in outdoor/indoor environments. J. Mater. Chem. A *8*, 17163–17173. 10.1039/d0ta04721j.

9. Wang, K.L., Li, X.M., Lou, Y.H., Li, M., and Wang, Z.K. (2021). CsPbBrI2 perovskites with low energy loss for high-performance indoor and outdoor photovoltaics. Sci. Bull. *66*, 347–353. 10.1016/j.scib.2020.09.017.

10. He, X., Chen, J., Ren, X., Zhang, L., Liu, Y., Feng, J., Fang, J., Zhao, K., and Liu, S. (Frank) (2021). 40.1% Record Low-Light Solar-Cell Efficiency by Holistic Trap-Passivation using Micrometer-Thick Perovskite Film. Adv. Mater. *33*, 2100770. https://doi.org/10.1002/adma.202100770.

11. Yang, F., Su, Z., Pascual, J., Li, M., Liu, H., Qin, C., Gao, X., Li, G., Li, Z., and Wang, Z. (2022). Enhancement of exciton separation in indoor perovskite photovoltaics by employing conjugated organic chromophores. J. Power Sources *520*, 230785. 10.1016/j.jpowsour.2021.230785.

12. Guo, Z., Jena, A.K., Takei, I., Ikegami, M., Ishii, A., Numata, Y., Shibayama, N., and Miyasaka, T. (2021). Dopant-Free Polymer HTM-Based CsPbI 2 Br Solar Cells with Efficiency Over 17 % in Sunlight and 34 % in Indoor Light. Adv. Funct. Mater *31*, 2103614. 10.1002/adfm.202103614.

13. Singh, R., Kumar, V., Parashar, M., and Sharma, V. (2022). Highly efficient quasi-cubic structured perovskite for harvesting energy from artificial indoor LED light source. Sol. Energy *245*, 332–339. 10.1016/j.solener.2022.09.015.

14. Raifuku, I., Ishikawa, Y., Chiang, Y.H., Lin, P.Y., Li, M.H., Uraoka, Y., and Chen, P. (2019).

Segregation-free bromine-doped perovskite solar cells for IoT applications. RSC Adv. *9*, 32833–32838. 10.1039/c9ra05323a.

15. Makming, P., Homnan, S., Ngamjarurojana, A., Rimjaem, S., Gardchareon, A., Sagawa, T., Haruta, M., Pakawatpanurut, P., Wongratanaphisan, D., Kanjanaboos, P., et al. (2023). Efficient and Stable Carbon-Based Perovskite Solar Cells Enabled by Mixed CuPc:CuSCN Hole Transporting Layer for Indoor Applications. ACS Appl. Mater. Interfaces *15*, 15486–15497. 10.1021/acsami.2c23136.

16. Penpong, K., Seriwatanachai, C., Naikaew, A., Phuphathanaphong, N., Thant, K.K.S., Srathongsian, L., Sukwiboon, T., Inna, A., Sahasithiwat, S., Pakawatpanurut, P., et al. (2023). Robust perovskite formation via vacuum thermal annealing for indoor perovskite solar cells. Sci. Rep. *13*, 10933. 10.1038/s41598-023-37155-4.

17. Pitchaiya, S., Eswaramoorthy, N., Madurai Ramakrishnan, V., Natarajan, M., and Velauthapillai, D. (2022). Bio-Inspired Graphitic Carbon-Based Large-Area (10 × 10 cm^2) Perovskite Solar Cells: Stability Assessments under Indoor, Outdoor, and Water-Soaked Conditions. ACS Appl. Mater. Interfaces *14*, 43050–43066. 10.1021/acsami.2c02463.

18. Passatorntaschakorn, W., Khampa, W., Musikpan, W., Cergy, C.Y., and Universit, P. (2023). A novel carbon electrode for up-scaling flexible perovskite solar cells. Appl. Mater. Today *34*, 101895. 10.1016/j.apmt.2023.101895.
