# Supplementary material for: Cs and Br tuning to achieve ultralow-hysteresis and high-performance indoor triple cation perovskite solar cell with low-cost carbon-based electrode
Source: iScience. 2024 Feb 22;27(4):109306. doi: 10.1016/j.isci.2024.109306 (PMC10940937; doi:10.1016/j.isci.2024.109306)
Supplement: Data S1. Standardized data reporting for photovoltaic cells [file mmc2.pdf]

Standardized data reporting for photovoltaic cells

CellPress

Revision 1.0, Last updated: Sept 11, 2020

If a key point of your paper is the performance of a photovoltaic cell, complete the below form

» Authors are encouraged to include this completed document as supplemental information at the time of submission. Not doing so may cause delays if your paper is selected for peer review.

» Please confirm that each item in the list is present in the manuscript or supplemental information by checking the corresponding box. For any items that are not present, please provide details in the text box for the editorial team and reviewers to consider.

Performance reporting

Item	confirm	Explain exception if line item is not present in manuscript
Current-voltage characteristics plotted in both forward and reverse directions	☑	
Current-voltage scan speed and dwell time	☑	
Details of device preconditioning, if any, prior to testing *for example light soaking or holding cell at a bias	☑	
Testing environmental conditions *such as atmosphere, temperature, and humidity	☑	
Statistical breakdown of performance across multiple devices *including number of individual substrates tested	☑	
Performance of any control devices are benchmarked to literature	☑	
Details of antireflection coating, if used	☑	There is no antireflection coating in this work.

Cell area

Item	confirm	Explain exception if line item is not present in manuscript
Device contact area	☑	
Device illuminated area during testing	☑	
How contact and illumination area were determined, including mask/aperture details	☑	
Performance reporting at 1 cm^2 cell *highly desirable but not mandatory	☑	

Device stability

Item	confirm	Explain exception if line item is not present in manuscript
Evolution of efficiency or current density (Jsc) at maximum power point *at least 100 seconds is recommended	☑	

Atmosphere, temperature, humidity, bias, illumination, and storage conditions for long-term testing	☑ confirm	
Encapsulation details, if used	☑ confirm	

Supporting characterization

External quantum efficiency (EQE) of key representative devices	☑ confirm	
Quantification of mismatch between integrated Jsc from EQE with Jsc from operational illumination source	☑ confirm	
Total reflectance or absorption spectra of cell illuminated from front side	☑ confirm	
Dark current-voltage characteristics shown on logarithmic current axis in both sweep directions	☐ confirm	
For tandem devices: EQE, bias voltage, and bias illumination of each subcell	☐ confirm	This work is not tandem device.

Performance verification

*highly desirable for performance claims near or exceeding state-of-art

Certification or other independent verification of performance results	☐ confirm	We have only in-house testing.

Calibration of illumination source and reference cell

Model of light source, reference cell, and other power sensors used in testing	☑ confirm	
Date when reference cell was last calibrated and certified	☐ confirm	
Spectral mismatch between reference cell and testing cells	☐ confirm	
Spectra of solar simulator used for measurement, including any additional filters used, and date it was measured	☐ confirm	

For further reading, see:

1. *Solar Cells Reporting Summary*. Nature Research. https://www.nature.com/documents/nr-photovoltaic-reporting.pdf
2. Reese et. al. *Reliably Measuring the Performance of Emerging Photovoltaic Solar Cells*. Nanostructured Materials for Type III Photovoltaics, 1–32 (2017).
3. Wang et. al. *Reliable Measurement of Perovskite Solar Cells*. Adv. Mater. 31, 1803231 (2019).
4. Timmreck et. al. *Characterization of tandem organic solar cells*. Nature Photon. 9, 478–479 (2015).
5. Kiermasch et. al. *Effects of Masking on Open-Circuit Voltage and Fill Factor in Solar Cells*. Joule 3, 16–26 (2019).
6. Fell. *Standardising current–voltage measurements for metastable solar cells*. JPhys Energy. 2, (2019).
7. ASTM. *Committee E44 on Solar, Geothermal and Other Alternative Energy Sources*.
8. IEC. *TC 82 Solar photovoltaic energy systems*.

I verify that this form is completed accurately in agreement with all co-authors, to the best of my knowledge ☑

Submitting author name: **Ladda Srathongsian**

For general queries or feedback regarding this form please email joule@cell.com
